# A set of gene knockouts as a resource for global lipidomic changes

**DOI:** 10.1038/s41598-022-14690-0

**Published:** 2022-06-22

**Authors:** Aleksandra Spiegel, Chris Lauber, Mandy Bachmann, Anne-Kristin Heninger, Christian Klose, Kai Simons, Mihail Sarov, Mathias J. Gerl

**Affiliations:** 1 Max Planck Institute of Molecular Cell Biology and Genetics, Pfotenhauerstrasse 108, 01307 Dresden, Germany; 2 Lipotype GmbH, Tatzberg 47, 01307 Dresden, Germany

**Keywords:** Biochemistry, Molecular medicine

## Abstract

Enzyme specificity in lipid metabolic pathways often remains unresolved at the lipid species level, which is needed to link lipidomic molecular phenotypes with their protein counterparts to construct functional pathway maps. We created lipidomic profiles of 23 gene knockouts in a proof-of-concept study based on a CRISPR/Cas9 knockout screen in mammalian cells. This results in a lipidomic resource across 24 lipid classes. We highlight lipid species phenotypes of multiple knockout cell lines compared to a control, created by targeting the human safe-harbor locus AAVS1 using up to 1228 lipid species and subspecies, charting lipid metabolism at the molecular level. Lipid species changes are found in all knockout cell lines, however, some are most apparent on the lipid class level (e.g., SGMS1 and CEPT1), while others are most apparent on the fatty acid level (e.g., DECR2 and ACOT7). We find lipidomic phenotypes to be reproducible across different clones of the same knockout and we observed similar phenotypes when two enzymes that catalyze subsequent steps of the long-chain fatty acid elongation cycle were targeted.

## Introduction

The phenotypic omics sciences are expanding to explore how gene expression, proteins, and metabolites are varying in different metabolic states. A major challenge for the omics sciences is to investigate how different metabolic pathways are regulated in health and disease. The process to identify the genomic bases of diseases and connect them to protein and enzyme malfunctions is already well established. A considerable knowledge base has been accumulated to document which enzymes are responsible to generate and regulate different metabolites. However, the field of lipidomics is still far away from defining enzyme specificity. So far, the classical work of lipid metabolism charted how lipid classes are metabolized. But this is insufficient for understanding pathologies at the molecular level and identifying diagnostic markers and/or therapeutic targets. Lipids are hydrophobic metabolites, which are built from a defined set of building blocks. Some of these, most predominantly the fatty acids, have different carbon lengths, numbers and positions of double bonds, and additional modifications such as hydroxylations. The number of possible combinations is estimated to have the potential to generate 9000–100,000 different molecular lipid species^1,2^. Although these molecular species can be quantitatively analyzed by different mass spectrometric methods, comprehensive knowledge of which enzymes and proteins are generating and metabolizing them is still lacking.

In this proof-of-concept study, we introduce a screening approach to chart lipid metabolism at the molecular level by combining lipidomic analysis with the CRISPR/Cas9 based knockout of genes involved in lipid metabolism in a mammalian cell line. While the data found in the literature are often not comparable due to the use of different starting material (e.g., different organs, cell lines, or derived from different organisms) and different lipid classes analyzed with different lipidomics platforms^3^, we explored how multiple gene knockouts in the human colorectal carcinoma (HCT116) cell line affect the lipidome, by building a consistent dataset.

Such a lipidomic screen is challenging due to the intricacies of lipid metabolism, in particular its redundancy regarding enzyme specificities as well as lipid functionalities. This redundancy is typically manifested as correlations between different groups of lipids sharing structural features^4^. First, if one protein is knocked out, other enzymes may have partially or completely overlapping activities and can compensate for the loss. Ceramides, for example, can be produced in a de novo pathway, but also by the salvage pathway^5^. Second, the functions of specific lipids can be taken over by other lipids. Therefore, when a cell lacks the ability to produce a given lipid, the activation of the synthesis of lipids in a different metabolic pathway can compensate for the loss and result in secondary phenotypic effects, so-called ripple and compensatory effects^6^. Third, the lipidome changes depending on the developmental stage of a cell potentially masking phenotypes or generating secondary phenotypic effects^7–9^.

Despite these challenges, in this proof-of-concept study, we find lipidomic phenotypes to be reproducible in replicates and across different clones. Particularly noteworthy is the fact that this was also true when two subsequent enzymes of the same metabolic pathway were knocked out. We are providing a resource that will serve as a starting point for establishing links between enzyme activities and their influence on the molecular lipid composition by measuring quantitative and multiparametric lipidomic phenotypes. Thus, we provide information on enzyme specificity and chart lipid metabolism at the molecular level.

## Results

### Generation and validation of knockout lines

**Figure 1:**
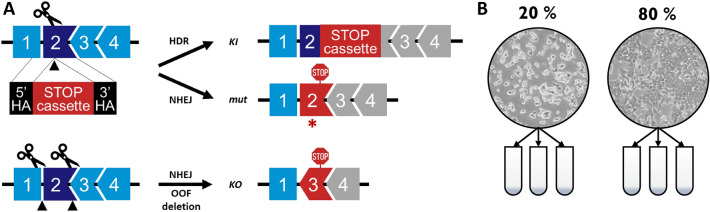
Sample preparation: (**A**) A schematic illustration of the strategies for CRISPR/Cas9 mediated generation of constitutive gene knockouts in HCT116 cells. Upper panel: Single guide RNA targeting an early exon of a gene of interest will generate a double-strand break (DSB). In the presence of a DNA repair donor containing a promoterless gene trap (stop) cassette flanked with 5’ and 3’ in-frame homology arms (HA) two DSB repair outcomes are possible: (1) homology-directed repair (HDR) will result in an in-frame knockin (KI) of the stop cassette leading to gene inactivation or (2) the non-homologous end joining (NHEJ) repair will generate INDEL mutation (mut, represented by an asterisk), leading to a premature stop codon (red stop sign). Lower panel: targeting introns flanking an early critical exon with two guide RNAs will lead to an out-of-frame (OOF) deletion of the critical exon. Following the NHEJ-mediated repair, a premature stop codon (red stop sign) will be created in the downstream coding sequence, leading to gene knockout (KO). (**B**) KO cell lines were grown to densities of 20% and 80% confluency and samples were collected in triplicates.

To generate permanent loss-of-function HCT116 cells for non-essential genes known or predicted to be involved in lipid metabolism we decided on a targeted genome engineering strategy, employing a CRISPR/Cas9 mediated gene trap (stop) cassette insertion (Fig. 1A), as previously described^10^ (*gene trap method*). The insertion of the stop cassette was confirmed by PCR and Sanger sequencing (Suppl. Table S01). Only the following two types of cell lines with a biallelic knockout were considered for further analysis: lines where both alleles were targeted by the stop cassette (referred to in the figures and text as KI/KI, knockin/knockin), and lines, in which one of the alleles was disrupted by the stop cassette insertion, while the other was disrupted by the error-prone non-homologous end joining (NHEJ) repair mechanism leading to an INDEL mutation and to a premature stop codon (referred to as KI/mut, See Fig. 1A). However, for 3 genes (ALDH3A2, CERS2, FADS3) no knockout clone could be obtained with this approach. Therefore, for these genes we used two guideRNAs to create larger deletions, that would either cause a frameshift or lead to a truncation of the catalytic domain (*deletion method*). Both methods result in a loss-of-function. For 2 genes, CEPT1 and GNPAT, both approaches were used for comparison and validation of the lipidomic result. One or more independent knockout lines were successfully obtained for **23** genes (Table 1). The gene targeting designs, guideRNAs used, and validation data for each individual gene are listed in (Suppl. Table S01 and Table S02). As a control cell line, we inserted the trap cassette at the human safe-harbor locus Adeno-Associated Virus Integration Site 1 (AAVS1).

**Table 1:** Overview of knockout methods, genotypes, and cell lines created, as indicated by the 23 knocked out genes as well as AAVS1 control and WT cell lines.

Method	Description	Genes
	WT	WT (1)
Gene trap	KI/mut	AAVS1_Con (1), KMT2C (1), LPIN1 (1), NSUN3 (1), PLD3 (1), THUMPD3 (1), ZDHHC5 (1)
Gene trap	KI/KI	ACOT7 (1), ACSL3 (2), CEPT1 (1), CERS2 (1), DECR2 (1), DLAT (2), ELOVL5 (1), GNPAT (1), HSD17B12 (1), KMT2C (1), MPDU1 (1), NSUN2 (1), ORMDL2 (2), SAMD8 (1), SGMS1 (1), ZDHHC12 (2)
Deletion	KO/KO	ALDH3A2 (2), CEPT1 (2), CERS2 (2), FADS3 (1), GNPAT (1)

The number of clonal cell lines are indicated in parentheses.

Knockout lines were grown in triplicates to a confluency of 20% and 80% (Fig. 1B), after which the lipidomes were analyzed. One or more independent knockout lines were successfully obtained for **23** genes resulting in a total of **36** clonal cell lines and 194 lipidomic measurements (Table 1).

### Lipidomics overview

**Figure 2:**
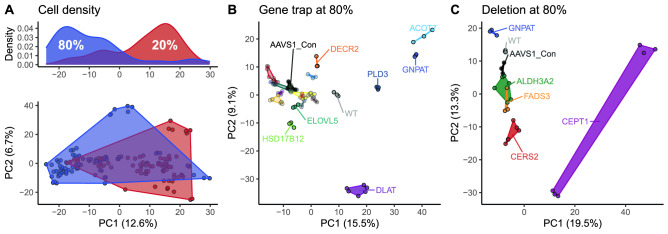
Principal component analysis: (**A**) Lower panel: Score plot of a PCA on all lipids occurring in every replicate of at least one cell line and density (n = 1809), colored by cell density, i.e., 20% or 80% confluency in the petri dish. Missing values were imputed with the median and only samples for which both densities were available were used. Percentages of variability explained in each principal component are indicated in the axis labels. Upper panel: Density of cell density in samples along principal component 1 (PC1). (**B**) PCA score plot of the PCA using all lipids occurring in every replicate of at least one cell line (n = 1449). Only samples that were generated at 80% confluency with the gene trap method are used. Samples are colored by gene targeted. Note that some genes are represented with one clone (a total of 3 replicates), while other genes are represented by two clones (a total of 6 replicates). Genes well separating from the bulk and control samples are highlighted and named. The percentage of variability explained in each principal component is indicated in the axis labels. (**C**) Same as B (n lipids = 1590) but containing samples that were generated with the deletion method.

Lipidomics analysis resulted in 2095 lipid species within 24 lipid classes. To get an overview of the lipidomes of all samples measured in the screen, we performed a principal component analysis (PCA). This indicated that the major variation in the screen was due to cell density on the culture dish (Fig. 2A). Because the different knockout lines had different growth patterns, the more reproducible and reliable 80% confluency was investigated, and we continued with clones from this cell density only. We performed a new PCA on only 80% confluency samples, separated into the two rounds of knockout creation, which had also separate controls (Fig. 2B,C). Knockout cell lines appear in defined clusters, most of which appear close to the safe-harbor control line (AAVS1), implying that there are only small changes to their lipidomes. In addition to the enzymes known to be involved in lipid biosynthesis, some genes were included, which are apparently not involved in lipid biosynthesis (KMT2C, THUMPD3, NSUN2, NSUN3), or only marginally connected to lipids, such as protein palmitoyltransferases (ZDHHC12, ZDHHC5) (Suppl. Table S05). These can be used to estimate how knockouts, unrelated to lipid metabolism would perform. Expectedly, most of the knockout cell lines clustered close to the AAVS1 cell line in the PCA (Fig. 2B) and hierarchical clustering (Suppl. Figure S01A). However, some genes appear to also show a lipidomic effect, which might be an indication of a, possibly indirect, connection to lipid metabolism.

The replicates of some KO lines form distinct clusters at a greater distance to the control, implying strong and reproducible changes. This is also largely true for KO cell lines, for which we had generated more than one clone, e.g., CERS2, which indicates that the variation between the clones is smaller than the variation induced by the knockout. Exceptions to this are the two CEPT1 clones, as discussed below. Similarly, when we look at the coefficients of variation (CV) of lipid species *within* each gene knockout (across clones and replicates), we find the median CV at 13% for 80% confluency, while the median CV of the lipid species *across* all knockout cells lines shows a higher variation of 49% (Suppl. Table S03).

A heatmap with hierarchical clustering (Suppl. Figure S01) of all samples at 80% confluency results in a picture similar to the PCA clustering, with most replicates and cell lines of the same knockout clustering together, an effect which is more pronounced for lipid species (Suppl. Figure S01 A, C) than for classes (Suppl. Figure S01 B, D). The heatmaps also provide an overview of each cell line's changes compared to the total dataset.

### Individual gene KOs

Lipidomes of gene knockout cell lines were always compared to cells, which have also been subjected to the full knockout procedure by inserting the gene trap cassette in the safe-harbor locus (AAVS1) without disrupting any gene. This locus is both transcriptionally active and its disruption is hypothesized not to lead to discernible phenotypic effects^11^. However, we do see changes between the wild-type cell line and the AAVS1 control, which was selected in the same way as all other clones (Fig. 1). We find an about 15% decrease of PE in the AAVS1 control, while many other lipid classes increase significantly, e.g.: DAG, PA, PC O-, PE O-, PI, PS (Suppl. Figure S02). The differences between the untreated wild-type cells and the cells targeted at the AAVS locus may reflect the adaptation of the lipidome to the stress of electroporation, G418 selection, and clonal expansion. These changes highlight the importance of choosing the AAVS1 cell line as the control in comparisons to knockout cell lines. Although we are measuring 2095 lipid species in the full dataset, not all species are found in every sample. In fact, only a limited number of species are found in every single sample (Suppl. Table S03). Due to this fact, we are adjusting the number of lipid species for each comparison in a range of 456 to 1228 (Suppl. Table S05) as explained in the methods section. This approach was chosen to catch rare lipid species, which might only appear in a specific knockout, e.g. accumulate due to the blocked enzymatic reaction by the knockout. The numbers used and the results of all statistical tests comparing knockout cell lines to AAVS1 controls are provided in Suppl. Table S05.

**Figure 3:**
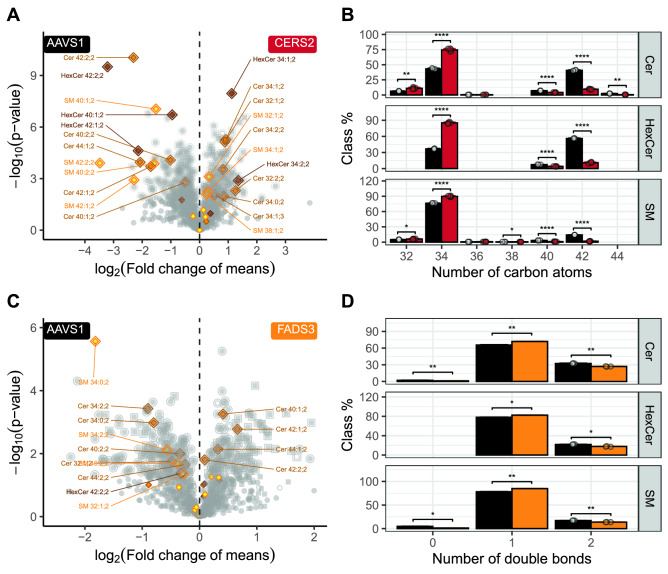
CERS2 and FADS3 Knockouts: (**A**) CERS2 KO Volcano plot: Lipid species of the CERS2 KO cell line (n = 6) and the AAVS1 control (n = 3) were compared. P-values of t-test without correction for multiple testing are displayed on the y-axis, fold changes of means are shown on the x-axis. Points with additional outlines indicate lipids significant after correction for multiple testing (Benjamini–Hochberg, 365 lipid species or 29.7% of all species). Sphingolipid species are labeled by their species names. (**B**) CERS2 KO sphingolipid length distribution: Lipid species have been standardized to the lipid class and summed up by their total number of carbon atoms. Values for individual samples are shown by points and means are indicated by bar plots. Error bars correspond to standard deviations. P-values have been adjusted for the total number length features in all classes (n = 168) and are encoded as follows: * for q < 0.05, ** for q < 0.01, *** for q < 0.01,**** for q < 0.0001. Only lipid species were used, which had at least 2 valid measurements in each of the two cell lines (n = 1228). The CERS2 KO clones A10 and B11 are displayed, which were created by the deletion method. (**C**) FADS3 KO Volcano plot: Lipid species of the FADS3 KO cell line (n = 3) and the AAVS1 control (n = 3) were compared. P-values of t-test without correction for multiple testing are displayed on the y-axis, fold changes of means are shown on the x-axis. Points with additional outlines indicate nominal significant lipids (87 lipid species or 8.2% of all species). Sphingolipid species are labeled by their species names. (**D**) FADS3 KO sphingolipid double bond distribution: Lipid species have been standardized to the lipid class and summed up by their total number of double bonds. Values for individual samples are shown by points and means are indicated by bar plots. Error bars correspond to standard deviations. p-values have been adjusted for the total number db features in all classes (n = 113) and are encoded as follows: * for q < 0.05, ** for q < 0.01, *** for q < 0.01,**** for q < 0.0001. Only lipid species were used, which had at least 2 valid measurements in each of the two cell lines (n = 1064). The FADS3 KO clone C02 is displayed, which was created by the deletion method.

As an initial proof of concept, we chose a well-known target (Fig. 3): ceramide synthase 2 (**CERS2**). Ceramides are sphingolipids that are created in mammals from sphinganine or sphingosine by sphinganine N-acyl transferases, a family of six ceramide synthases (CERS)^12^. CERS2 synthesizes ceramides containing mainly C20–C26 fatty acids^13^. We observe that the knockout of CERS2 leads to the expected reduction in sphingolipid species consistent with these fatty acid lengths (total length of C38—C44, Fig. 3B) as has also been seen in lipidomics studies in mice^14,15^. In addition to the reduction of sphingolipids with C20–C26 fatty acids, we see an increase in sphingolipids species consistent with C16 fatty acids (C-34), which shows the tendency of the cell line to compensate the inflicted change. Besides this direct effect, we see also many additional changes that impact about a third of all lipid species (Fig. 3A), causing CERS2-KO to be very different from the AAVS1 control in the PCA (Fig. 2C) and highlight the importance to use the complete lipidome to assess changes to the entirety of lipid metabolism that result from the loss of a single protein.

In contrast to CERS2, the fatty acid desaturase 3 (**FADS3**) KO displays a more subtle phenotype. Here, sphingolipid species with a total of 2 double bonds are reduced (Fig. 3C). This is likely due to the lack of a sphingadiene (L-18;2;2) long-chain base, which is known to have a second double bond in addition to the usual Δ4E double bond found in sphingosine^16^. However, we also find several sphinganine (L-18:0;2) lipids reduced, which have no double bond at all, e.g., most prominently SM 34:0;2 (Fig. 3A). In total, this renders the double bond distribution to become less diverse and sphingolipids with one double bond become even more abundant than in the control (Fig. 3D).

**Figure 4:**
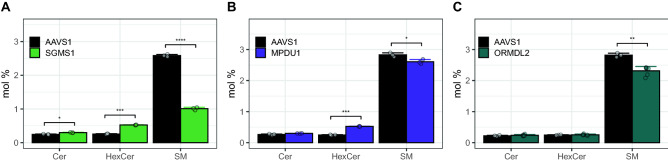
Sphingolipid Class effects: (**A**–**C**): Sphingolipid classes of the knockouts indicated compared to the AAVS1 control. (**A**) SGMS1 KO: See details in Suppl. Figure S06. (**B**, **C**) MPDU1 KO and ORMDL2 KO: See details in Suppl. Figure S07.

Further, we observed that different knockouts can have similar outcomes. The sphingomyelin synthase (**SGMS1)** catalyzes the transfer of phosphatidylcholine from PC onto Cer in the Golgi lumen (Suppl. Figure S06A), a reaction yielding sphingomyelin (SM) and diacylglycerol (DAG)^17^. Knocking out this enzyme resulted in a reduction of total SM levels to 34% as compared to the AAVS1 control. As the metabolic pathway from ceramide towards SM is blocked, there is a compensatory upregulation of the alternative pathway, because the total HexCer amount increases 1.7-fold (Fig. 4A). Interestingly under these conditions, the cholesterol level shows a dip, while cholesteryl ester (CE) levels more than double and ether-linked phosphatidylcholine (PC O-) levels are elevated (Suppl. Figure S06C). Besides these, there are multiple effects on the species level of the SGMS1 KO (Suppl. Figure S06B).

Similar to the SGMS1 KO phenotype, also in the Mannose-P-dolichol utilization defect 1 protein **(MPDU1)** KO a 2.1-fold increase in hexosyl ceramide (HexCer) and a 7% reduction of SM, as compared to the AAVS1 control, is found (Fig. 4B). In contrast, the **ORMDL2** KO only leads to an 18% reduction of SM (Fig. 4C).

**Figure 5:**
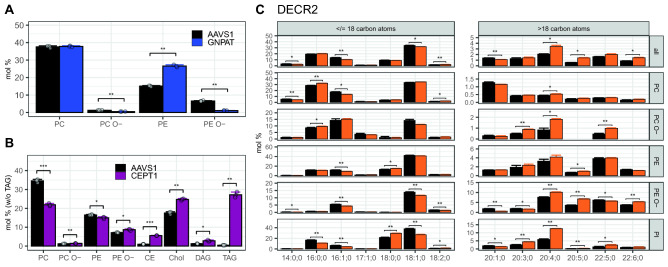
Genes in GP metabolism compared to AAVS1 control. (**A**) GNPAT KO—GP classes with choline and ethanolamine head groups: Diacyl (PC, PE) and ether-linked (PE O-, PC O-) lipid classes are shown. See details in Suppl. Figure S03 (**B**) CEPT1 KO—Lipid classes: Due to the strong differences in TAG levels, all lipids are standardized to the total lipid content without TAG. The CEPT1 KO clone B8 is displayed, which was created by the deletion method. See details Suppl. Figure S05. (**C**) DECR2 KO—DECR2 knockout: Lipid species of the DECR2 KO cell line (n = 3) and the AAVS1 control (n = 3) were compared. Fatty acid profile: greater than 1 mol% across CE, DAG, LPC, LPE, LPE O-, LPG, LPI, PA, PC, PC O-, PE, PE O-, PG, PI, PS, TAG lipid classes and individual profiles of PC, PC O-, PE, PE O-, PI. Means are indicated by bar plots. Error bars correspond to standard deviations. p-values have been adjusted for the total number fatty acids (n = 51) or the total number fatty acids and lipid classes (n = 263) and are encoded as follows: * for q < 0.05, ** for q < 0.01, *** for q < 0.01,**** for q < 0.0001. Only lipid species were used, which had at least 2 valid measurements in each of the two cell lines (n = 734). The DECR2 KO clone C05 is displayed, which was created by the gene trap method.

Lipid classes with ether-linked carbon chains are regulated differently than their corresponding fatty acid (diacyl-) based lipid classes^18^. Consistent with this, we observed that when **GNPAT** (a peroxisomal dihydroxyacetone phosphate acyltransferase, (Fig. 5A) is knocked out, ether lipids are strongly reduced. Total PE O- levels are lowered to just above 17% of the AAVS1 control (Fig. 5A), an effect which involved close to all subspecies levels in the GNPAT KO (Suppl. Figure S03D). Similarly, diacyl PE levels are significantly elevated (Fig. 5A), which also involves most PE species (Suppl. Figure S03B). We find no effect on total PC, while PC O- levels are reduced. However, the number of individual species of PC (Suppl. Figure S03C) and PC O- (Suppl. Figure S03E) show similar trends as the PE/PE O- pair. These results are based on the GNPAT KO clone, created by the gene trap method, and are largely replicated in the GNPAT KO clone, created by the deletion method (Suppl. Figure S04). This confirms the results with two independent cell lines created with two different techniques.

The Choline/ethanolaminephosphotransferase 1 (**CEPT1**) knockout cell line shows a strong reduction of total PC levels (Fig. 5B), also lowering levels of more than 80% of its subspecies (Suppl. Figure S05B).The effect on PE is less pronounced at the class level, and we only see only around 60% of PE subspecies levels reduced (Suppl. Figure S05C). Also, here, levels of ether-linked PC O- and PE O- behave in the opposite direction. Furthermore, in the CEPT1 KO, we find many very strong effects on multiple other lipid species (Suppl. Figure S05A) and classes, especially DAG, cholesterol, and the storage lipid classes: TAG and CE. For CEPT1, also two KO clones with similar effects were found, however, the magnitude of the phenotype varied significantly (Fig. 2; Suppl. Figure S05D). We validated the results with a third clone, generated by the deletion knockout method, and detected a very similar phenotype (Suppl. Figure S05E).

**Figure 6:**
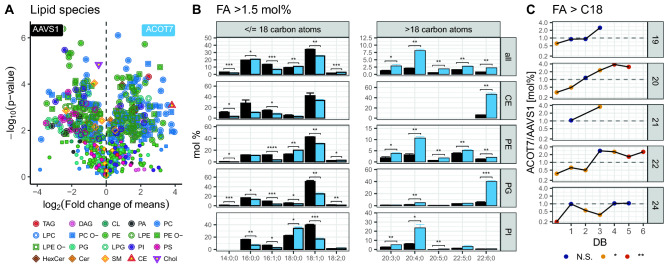
ACOT7 knockout: (**A**) Volcano plot: Lipid species of the ACOT7 KO cell line (n = 3) and the AAVS1 control (n = 3) were compared. P-values of t-tests without correction for multiple testing are displayed on the y-axis, fold-changes of means are shown on the x-axis. Points with additional outlines indicate lipids significant after correction for multiple testing (Benjamini–Hochberg, 358 lipid species or 54.1% of all species). The shapes and colors of points indicate the lipid class of the species. (**B**) Fatty acid profile: greater than 1.5 mol% across CE, DAG, LPC, LPE, LPE O-, LPG, PA, PC, PC O-, PE, PE O-, PG, PI, PS, TAG lipid classes and individual profiles of CE, PE, PG, PI. Means are indicated by bar plots. Error bars correspond to standard deviations. p-values have been adjusted for the total number fatty residues (n = 50) or the total number fatty residues and lipid classes (n = 240) and are encoded as follows: * for q < 0.05, ** for q < 0.01, *** for q < 0.01, **** for q < 0.0001. (**C**) Fatty acid ratios: of FAs greater than 18 carbon atoms across CE, DAG, LPC, LPE, LPE O-, LPG, PA, PC, PC O-, PE, PE O-, PG, PI, PS, TAG lipid classes. Fatty acid length in carbon atoms is shown in panels and fatty acid double bonds on the x-axis. The ratio of the means ACOT7/AAVS1 [mol%] as depicted in (**B**) is shown on the y-axis, with the corresponding p-values corrected for multiple testing encoded in color. Only lipid species were used, which had at least 2 valid measurements in each of the two cell lines (n = 662). The ACOT7 KO clone G12 is displayed, which was created by the gene trap method.

Some knockouts were mainly affecting fatty acid profiles. For example, for the acyl-CoA thioesterase (**ACOT7**), which catalyzes the hydrolysis of acyl-CoAs to free fatty acids and coenzyme A, differences in the overall fatty acid profiles are expected. Indeed, we find a lot of species differences (Fig. 6A) but also that in the KO cell line, total fatty acids with 20 or more carbon atoms are strongly increased (Fig. 6B), with some of the main affected lipid classes highlighted. In addition, we find double bond specificity in C20 to C22 fatty acids, which suggests ACOT7 activity towards unsaturated, but not saturated fatty acids (Fig. 6C).

Another example is the peroxisomal 2,4-dienoyl-CoA reductase (**DECR2)**, an auxiliary enzyme in beta-oxidation, which also participates in the degradation of unsaturated fatty enoyl-CoA esters^19^. Therefore, it is expected to see fatty acid profile changes, however, these are mostly found in levels of long-chain fatty polyunsaturated fatty acids in PI, PE O- and PC O- classes (Fig. 5C). This is particularly interesting, as the latter two ether-linked lipid classes are also synthesized within the peroxisome.

**Figure 7:**
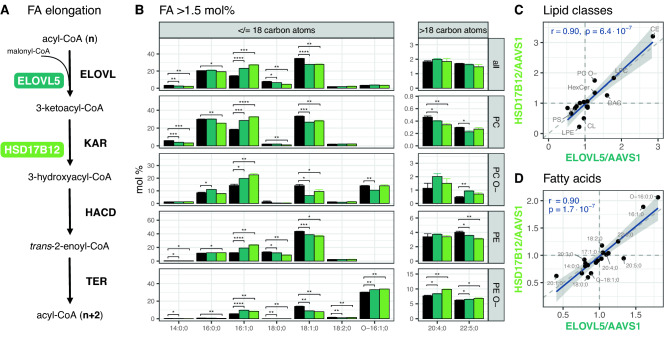
HSD17B12 and ELOVL5 knockouts compared to AAVS1 control (black). (**A**) The long-chain fatty acid elongation cycle consists of 4 enzymatic steps that in sequence extend the fatty acid (n) by two carbon atoms (n + 2). (1) Elongation of very-long-chain fatty acids (ELOVL 1–7); (2) 3-ketoacyl-CoA reductase (KAR); (3) 3-hydroxyacyl-CoA dehydratase (HADC); (4) trans-2,3-enoyl-CoA reductase (TER). (**B**) Fatty acid profiles: greater than 1.5 mol% across CE, DAG, LPC, LPE, LPE O-, PC, PC O-, PE, PE O-, PG, PI, PS lipid classes and individual profiles of PC, PC O-, PE, PE O-. Fill colors of ELOVL5 and HSD17B12 are indicated in (**A**). The AAVS1 control is shown in black. Means are indicated by bar plots. Error bars correspond to standard deviations (n = 3). p-values have been adjusted for the total number of fatty acids (n = 47) or the total number of fatty residues and lipid classes (n = 199) within each gene knockout and are represented by stars. Only lipid species were used, which appeared at least 2 × in each of the 3 cell lines (n = 517). (**C**) Correlation of lipid class ratios: of ELOVL5/AAVS1 and HSD17B12/AAVS1. The Pearson’s correlation coefficient (r) and its p-value are shown in the upper left corner. (**D**) Correlation of total FA ratios: same as (**D**) for total fatty acid sums.

Finally, we tested two genes involved in the same pathway. **ELOVL5** and **HSD17B12** are the first two genes of the long-chain fatty acid elongation cycle (Fig. 7A), which extends fatty acids by two carbon atoms^20,21^. Although knockout cell lines for both genes display some lipidomic difference, overall ELOVL5 and HSD17B12 show similarities by proximity in the PCA score plot (Fig. 2), hierarchical clustering (Suppl. Figure S01 A), and very similar fatty acid profiles within lipid classes (Fig. 7B). This similarity becomes even clearer when the ratio of lipid classes in the knockout cell lines to the AAVS1 control cell line was compared and showed a high degree of correlation (Fig. 7C). The same is true for the correlation of the ratios of total fatty acid levels between the knockout cell lines and the AAVS1 control (Fig. 7D). These similarities, especially in the fatty acid profiles, clearly show the power and reproducibility of gene knockouts when investigating enzyme specificity.

## Discussion

Investigating lipid enzyme specificity is difficult and detailed biochemical work. Here we present a proof-of-concept study, that uses CRISPR/Cas9-based approach to generate single-gene knockout cell lines and measures the whole lipidome of each. This contrasts with other CRISPR/Cas9-based lipidomics experiments, where only a few specific genes of interest are targeted and examined^22,23^. While genome-wide CRISPR/Cas9 screens exist in the literature, the method used was different from ours because the genome-wide libraries were applied to a pool of cells, which were subsequently treated to enrich and identify interesting candidates. These candidates were then investigated by MS-based lipidomics analysis^24,25^. In contrast, in our study, we isolate and expand knockout clones and determine their lipidome, because we are interested in how the cell adapts to the changes. Thus, we obtain information on how the adapted phenotype is reflected in the lipidome, describing how flexible the cellular lipidome is. We found it necessary to compare each knockout cell line to the AAVS1 control, which was subjected to the full treatment as the knockout cell lines, as this treatment already altered the lipidome, when compared to the WT strain. The genes, which gave viable knockouts in our screen are obviously not essential for the HCT116 cell line, although two of the genes (HSD17B12, LPIN1), were suggested to be essential in humans^26^. Different densities were initially used as we expected that this would influence the lipid composition^27,28^. This turned out to be true, however, as only the 80% density was robustly reproducible, we report this density in the detailed analyses.

In the **FADS3** KO, we see a shift in the double bond profile away from both, 0 and 2 double bonds, towards sphingolipids, which contain a total of 1 double bond. This fits well with the previously reported knockout and knockdown of FADS3, which was identified as a long-chain base Δ14Z desaturase by sphingolipid profiling^29,30^. Targeted methods like these are of course ideally suited to investigate this specific knockout. The mass spectrometry methods applied in this screen were designed for high throughput of samples and the identification of a large set of lipid species. With this method, we were not able to identify the 18:2;2 sphingoid bases in our spectra with enough confidence to report a sphingoid base profile. However, additional double bonds are easily detected on the species level as sphingolipids with fatty acids shorter than 24 carbon atoms rarely have any double bonds. Interestingly, the one species with 2 double bonds found to be slightly increased in the FADS3 KO is Cer 42:2;2, most likely containing a C-24 fatty acid. In human plasma this species consists of slightly more of the subspecies containing an unsaturated nervonic fatty acid (Cer 18:1;2/24:1;0, > 60%) than of the sphingadiene containing subspecies Cer 18:2;2/24:0;0 (< 40%)^31^. It, therefore, makes sense, that the reduction of double bonds on the LCB part is potentially compensated by the incorporation of an unsaturated fatty acid. The same argument, however, *cannot* be made for the reduction of sphingolipids with no double bond in the FADS3 KO, containing sphinganines and saturated fatty acids. The double bond missing compared to sphingolipids with 1 double bond, is not a *cis* double bond located in the hydrophobic core of the membrane, as it is the case for above mentioned nervonic acid (Δ15Z) and sphingadiene (Δ14Z), which increase membrane fluidity. But the missing double bond is the Δ4 *trans* double bond located in the polar region of the membrane, which has been reported to increase membrane packing^32^. Although it is counter-intuitive to decrease membrane fluidity even more as a consequence of a process that already does exactly this, similar results have been reported in human plasma, where increased sphinganine long-chain base levels (16:0;2 and 18:0;2) were found in women, which also have higher FADS3 expression in many tissues and higher sphingadiene levels in plasma^33^.

In the **SGMS1** KO, we see the expected reduction of total sphingomyelin, while Cer and HexCer levels are increased due to backlog or flow into an alternative pathway. This has been previously reported in lipidomic studies of SM synthase 1/2 double KO mouse embryonic fibroblasts^34^. However, we also see a dip in the cholesterol level, which is likely related to the SM-sequestered cholesterol pool in the plasma membrane^35^. Similarly, a coordinated regulation of ether lipids (i.e., PC O-) with sphingolipids has been shown^36^, in which reduction of sphingolipids with myriocin leads to an increase of PC O-, similar to what we have seen in SGMS1 KO cells. A similar phenotype as in the SGMS1 KO is observed in the **MPDU1** KO. Surprisingly, MPDU1 is required for efficient use of Dol-P-Man and Dol-P-Glc in mammalian cells and the synthesis of N-linked and O-linked oligosaccharides and GPI anchors. How this results in a 1.7-fold increase of HexCer and reduction of SM to 80% compared to the AAVS1 control is unknown. However, it is tempting to speculate that by blocking the protein glycosylation more sugar building blocks are available, which eventually would increase the amount of HexCer on the cell surface.

**ORMDL** proteins (ORMDL1, 2, and 3) inhibit the de novo synthesis pathway of sphingolipids by inhibiting serine palmitoyl transferase^37^, in response to sensing elevated ceramide levels^38^. ORMDL proteins are redundant and might be essential for maintaining control of the de novo sphingolipid biosynthesis when there is a high demand for sphingolipids^39^. Although ORMDL protein knockouts should increase sphingolipid levels, **ORMDL2** KO does not do so in mice^39^. Surprisingly, in our ORML2 KO, we find no elevation of sphingolipids as expected for the knockout of a sphingolipid inhibiting factor but again a significant reduction of sphingomyelin, indicating that further studies are needed to understand the function of ORMDL proteins.

In the case of glycerophospholipids, we studied a series of knockouts, which are characterized by changes in lipid class levels: **GNPAT** is a peroxisomal dihydroxyacetone phosphate acyltransferase (DAP-AT, DHAP-AT)^40^, which catalyzes the first step in plasmalogen biosynthesis^41^. The KO of GNPAT in mice results in a model for the severe congenital disorder rhizomelic chondrodysplasia punctata due to a lack of plasmalogens^42^. GNPAT is not the rate-limiting, however, an essential step of ether-lipid biosynthesis^18^, in agreement with this, we find ether lipids in the GNPAT KO cell line reduced. We clearly find total PE O- strongly reduced, while diacyl PE was increased. CRISPR knockouts of GNPAT in a Jurkat cell line showed a stronger reduction of PC O- lipids than for PE O-, highlighting cell line specific effects^43^.

**CEPT1** catalyzes the terminal step of the Kennedy Pathway^44^ for both PC and PE from diacylglycerol (DAG) and CDP-choline and CDP-ethanolamine, respectively^45^. Our KO cell line shows a strong reduction of PC levels and a lesser effect for PE. Levels of ether-linked PC O- and PE O- are even increased, although in in vitro mixed micellar assays specificity for diradylglycerol containing ether linkages was demonstrated^46^. In line with DAG being a substrate to the blocked reaction, higher DAG levels are found. However, the much higher TAG and CE levels in CEPT1 KO are likely explained by CEPT1-derived PC “16:0/18:1-GPC “ as an essential ligand for PPARα activation of β-oxidative lipid metabolism^47^. In agreement with this, we find PC 16:0;0_18:1;0 to be reduced to 55% in the CEPT1 KO cell line compared to controls (p(BH) = 0.001). The fact that we isolated two different clones with similar phenotypes but different intensity, shows that, although clonal variability was overall small compared to the phenotypes observed, it is a factor to be considered.

Some general effects we found to revolve around the biosynthesis and degradation of fatty acids. **ACOT7** is an acyl-CoA thioesterase that catalyzes the hydrolysis of acyl-CoAs to the free fatty acid and coenzyme A with a broad specificity of chain lengths of C8-C18. ACOT7 may have important functions in the brain^48^ and gastric cancer progression^49^. Further, it has been recently implicated in fatty acid metabolism modulating lipid signaling in pancreatic β-cells^50^ and macrophages^51^. Although a knockout of ACOT7 should lead to more C8 to C18 acyl-CoA and their increased incorporation into complex lipids, we find these fatty acid lengths decreased and unsaturated fatty acids with C20 to C22 length strongly increased. Previously, overexpression of ACOT7 in pancreatic β-cells indeed lowered acyl-CoA levels, consistent with the reported C8 to C18 specificity^50^. However, ACOT7-deficient macrophage lysates also indicated that endogenous ACOT7 contributes little to total acyl-CoA thioesterase activity towards shorter acyl-CoA species (16:0, 18:1), but elevates C20:4-, C20:5-, and C22:6-CoA^51^. This is exactly what we also find in ACOT7 KO in the HCT116 cell line in this study, especially in FA 20:4 (3.8 × total increase) and FA 22:6 (> 6 × increase in PG and CE). Furthermore, we report a double bond specificity in C20 to C22 fatty acids, which suggests ACOT7 activity towards unsaturated, but not saturated fatty acids. This has been reported before^51–53^, but until now not been shown in complex lipids. Previous over-expression results suggest that ACOT7 has also activity towards C8 to C18 acyl-CoA^51^, but this might be compensated by other ACOT activities. There are 15 known acyl-CoA thioesterases in mice, each with differential tissue and intracellular localization, transcriptional regulation, and acyl-CoA substrate preference^51^.

In the degradation cycle of fatty acids, **DECR2** is an auxiliary enzyme of the peroxisomal beta-oxidation and participates in the degradation of unsaturated fatty enoyl-CoA esters having double bonds^19^. Although beta-oxidation is occurring primarily in mitochondria (with the help of a homologous DECR1 protein), the peroxisome can also shorten very long-chain fatty acids (up to C26) and then hand them over to the mitochondrial process, which is known to shorten long, medium, and short-chain fatty acids (< C18)^54,55^. In the DECR2 KO, we mostly see elevated levels of long-chain polyunsaturated fatty acids in PI, PE O- and PC O- classes, the latter two of which are also synthesized within the peroxisomes. This confirms earlier results that DECR2 can also act on 2-trans,4-trans-dienoyl-CoA^56^, but its main role is likely to assist in the degradation of PUFAs, e.g., arachidonic acid (20:4;0) and docosahexaenoic acid (22:6;0) in peroxisomes^57^.

Eventually, fatty acids must be synthesized de novo by the fatty acid synthase^58^ or derived from the diet. However, many fatty acids must also be elongated into long-chain and very long-chain fatty acid versions by specific membrane-bound enzymes localized in the endoplasmic reticulum. The long-chain fatty acid elongation cycle consists of 4 enzymatic steps that in sequence extend the fatty acid by two carbon atoms^20,21^. The first rate-controlling step is the condensation of a fatty acyl-CoA with malonyl-CoA catalyzed by enzymes named Elongation of very-long-chain fatty acids (ELOVL). Of the seven ELOVL enzymes with different specificities, **ELOVL5** was included in our study. This enzyme has a reported specificity for fatty acids with C-16, C-18, and C-20 carbon lengths^59–61^. In the second step, the product of the condensation reaction is reduced by 3-ketoacyl-CoA reductase (KAR). Its gene, termed ***HSD17B12,*** was also knocked out in our screen. Lipidomics analysis of blood serum of a mouse KO showed accumulation of several sphingolipid classes with shorter than 18-carbon fatty acid side chains, in line with the proposed role for HSD17B12 in fatty acid elongation^62^. HSD17B12 has been reported to have specificity for C-18, C-20, and C-22 carbon atoms length^63,64^. This corresponds exactly to the fatty acid length specificity of ELOVL5 after 2 carbon atoms have been added by its activity. The finding that these two genes with their predicted matching specificities and sequential activity within the same pathway create very similar results in our study, underscores the reliability of the lipidomic screening approach.

There are also knockouts in this study, the results of which do not confirm expectations, or their phenotype could not be explained. These data are included as a resource for future discovery or confirmation. Some of which, profoundly alter the lipidome, e.g., **PLD3**, which catalytic phospholipase D activity is still controversial^65,66^, while some only have only mild effects, e.g., ALDH3A2 or LPIN1. These mild phenotypes might be caused by several reasons, e.g., the enzymes not being active in the cell line or overlapping specificity with other enzymes.

Overall, the major value of this study lies in the approach of examining *multiple* gene knockouts in the same cell line measured with a *single* lipidomic approach, even though some of the genes in this study have been examined in other circumstances with MS-based lipid analysis. Only in our unified approach, the lipidomes in the different knockouts are comparable. In addition, for many of the previous studies that performed lipidomic analysis, referenced above, the actual lipidomic data are not included and therefore, not accessible. In contrast, we provide the full dataset on (sub-) species level to the community, creating a valuable resource for future investigations.

There is of course room for improvement in future screens to eliminate clonal effects^23^, and to improve the signal induced by the knockout over the noise created by e.g. cell density effects^27,28^ and effects of cell culture conditions^23,33,67^. Also, the HCT116 cell line we used, is a very suitable cell line for a screen, due to the ease of maintenance and editing, as well as the near-normal karyotype (unlike many immortalized lines) as well as the previously established methods in the same line^68^. However, each cell line presents a unique lipid metabolism and lipidome, mainly, because of their parent tissues, which already differ in lipid metabolic pathways^69^. The effects introduced by the knockouts might therefore also differ in other contexts.

## Conclusions

This proof-of-concept study shows that quantitative lipidomics screens are possible, produce reliable and reproducible data, and should be expanded in terms of gene numbers, throughput, and the use of different cell lines. Eventually, such screens will make it possible to create a map of all lipid pathways at the molecular level, incorporating the proteins involved at each different step of metabolism. Such comprehensive charting of lipid metabolism at the molecular level will provide a valuable resource for innovative diagnostic approaches and novel therapeutic targets.

## Materials and methods

### Creation of knockout cell lines

The HCT116 cell line used was obtained from the European Collection of Authenticated Cell Cultures (ECACC) via Sigma-Aldrich. The majority of the knockout cell lines were generated as described^10^ by an in-frame insertion of a promoter-less stop cassette encoding the G418 resistance and a stop signal into one of the gene of interest’s early exons, which are shared by all the gene’s known isoforms. CRISPR guide RNAs were selected based on low off-target and highest on-target activity using Geneious Prime software (version R11) and http://crispor.tefor.net. The guideRNAs were purchased as crRNA from Integrated DNA Technologies (IDT), complexed with tracrRNA (IDT) and HiFi Cas9 protein (IDT) The ribonucleoprotein (RNP) complex was subsequently electroporated into the HCT116 cells together with the DNA repair PCR product providing the donor template as dsDNA. The DNA donor comprised the stop cassette sequence flanked with 50 or 150 bp homology arms. Following electroporation and antibiotic selection, G418-resistant clones were isolated by single-cell sorting, followed by clonal expansion and genotyping by PCR and Sanger sequencing (Suppl. Table S01). Six genes were knocked out using an alternative method by generating a frameshift deletion (ALDH3A2, CEPT1, CERS2, GNPAT, SGMS1) or by truncation of the functional domain (FADS3). The deletion was generated using two paired CRISPR guide RNAs flanking the target sequence. Due to the lack of a selection marker and to increase the chance to obtain a knockout, two independent guide RNA pairs were designed for each gene (referred to as condition A and condition B, Suppl. Table S02).

The safe-harbor control line (AAVS1) was generated by inserting the stop cassette in the AAVS1 locus using the guide RNA and AAVS1 homology arm sequences as described by^70^ in https://www.nature.com/articles/nbt.3198. Following the electroporation, the cells were treated in the same way and the KO samples described above. The goal was to generate a control that would account for any changes in the lipid profile that might have resulted from the CRISPR/Cas9 procedure and antibiotic selection.

The HCT116 and derived cell lines were grown in McCoy’s 5A-GlutaMAX™ medium (Gibco) supplemented with 10% Fetal Bovine Serum (FBS). For selection and maintenance of G418-resistant clones, growth medium containing 250 μg/mL G418 (Sigma-Aldrich) was used.

To prepare the knockout clones for lipidomic analysis, the cells were grown in triplicates to confluencies of 20% and 80%, washed with PBS and trypsinised. 1.5 × 10^5^ cells were harvested for each sample, washed once with cold PBS, resuspended in 150 μl cold PBS, snap-frozen in liquid nitrogen, and stored at -80 °C prior to lipidomic analysis.

### Lipid nomenclature

Lipid molecules are identified as species or subspecies. Fragmentation of the lipid molecules in MSMS mode delivers subspecies information, i.e., the exact acyl chain (e.g., fatty acid) composition of the lipid molecule. MS-only mode, acquiring data without fragmentation, cannot deliver this information and provides species information only. In that case, the sum of the carbon atoms and double bonds in the hydrocarbon moieties is provided. Lipid species are annotated according to their molecular composition as Lipid class < sum of carbon atoms > : < sum of double bonds > ; < sum of hydroxyl groups > . For example, PI 34:1;0 denotes phosphatidylinositol with a total length of its fatty acids equal to 34 carbon atoms, a total number of double bonds in its fatty acids equal to 1, and 0 hydroxylations. In the case of sphingolipids, SM 34:1;2 denotes a sphingomyelin species with a total of 34 carbon atoms, 1 double bond, and 2 hydroxyl groups in the ceramide backbone. Lipid subspecies annotation contains additional information on the exact identity of their acyl moieties and their *sn*-position (if available). For example, PI 18:1;0_16:0;0 denotes phosphatidylinositol with octadecenoic (18:1;0) and hexadecanoic (16:0;0) fatty acids, for which the exact position (*sn*-1 or *sn*-2) in relation to the glycerol backbone cannot be discriminated (underline “_” separating the acyl chains). On contrary, PC O-18:1;0/16:0;0 denotes an ether-phosphatidylcholine, where an alkyl chain with 18 carbon atoms and 1 double bond (O-18:1;0) is ether-bound to the *sn*-1 position of the glycerol and a hexadecanoic acid (16:0;0) is connected via an ester bond to the *sn*-2 position of the glycerol (slash “/” separating the chains signifies that the *sn*-position on the glycerol can be resolved). Lipid identifiers of the SwissLipids database^71^ (http://www.swisslipids.org) are provided in Suppl. Table S03.

### Analytical process design

Samples were divided into analytical batches of 84 samples each. Each batch was accompanied by a set of 4 blank samples (150 mM ammonium bicarbonate (in water)) and a set of identical 8 control reference samples (human full blood plasma). These control samples in groups of 1 blank and 2 reference samples were distributed evenly across each batch and extracted and processed together with study samples to control for background and intra-run reproducibility.

### Lipid extraction for mass spectrometry lipidomics

Mass spectrometry-based lipid analysis was performed as described^72–75^. For all other types of samples, the procedure was as follows. Lipids were extracted using a two-step chloroform/methanol procedure^6^ with chloroform:methanol 10:1 (V:V) and 2:1 (V:V) in the first and second step, respectively^7^. Prior to extraction, samples were spiked with an internal lipid standard mixture containing: cardiolipin 16:1/15:0/15:0/15:0 (CL, 50 pmol per extraction), ceramide 18:1;2/17:0 (Cer, 30 pmol), diacylglycerol 17:0/17:0 (DAG, 100 pmol), hexosyl ceramide 18:1;2/12:0 (HexCer, 30 pmol), lyso-phosphatidate 17:0 (LPA, 30 pmol), lyso-phosphatidylcholine 12:0 (LPC, 50 pmol), lyso-phosphatidylethanolamine 17:1 (LPE, 30 pmol), lyso-phosphatidylglycerol 17:1 (LPG, 30 pmol), lyso-phosphatidylinositol 17:1 (LPI, 20 pmol), lyso-phosphatidylserine 17:1 (LPS, 30 pmol), phosphatidate 17:0/17:0 (PA, 50 pmol), phosphatidylcholine 17:0/17:0 (PC, 150 pmol), phosphatidylethanolamine 17:0/17:0 (PE, 75 pmol), phosphatidylglycerol 17:0/17:0 (PG, 50 pmol), phosphatidylinositol 16:0/16:0 (PI, 50 pmol), phosphatidylserine 17:0/17:0 (PS, 100 pmol), cholesterol ester 20:0 (CE, 100 pmol), sphingomyelin 18:1;2/12:0;0 (SM, 50 pmol), triacylglycerol 17:0/17:0/17:0 (TAG, 75 pmol) and cholesterol D6 (Chol, 300 pmol). After extraction, the organic phase was transferred to an infusion plate and dried in a speed vacuum concentrator. 1^st^ step dry extract was resuspended in 7.5 mM ammonium acetate in chloroform/methanol/propanol (1:2:4, V:V:V) and 2^nd^ step dry extract in 33% ethanol solution of methylamine in chloroform/methanol (0.003:5:1; V:V:V). All liquid handling steps were performed using Hamilton Robotics STARlet robotic platform with the Anti Droplet Control feature for organic solvents pipetting. All solvents and chemicals used were of analytical grade.

### MS data acquisition

Mass spectrometry analysis was performed as described previously for plasma^73^. Samples were analyzed by direct infusion in a QExactive mass spectrometer (Thermo Scientific) equipped with a TriVersa NanoMate ion source (Advion Biosciences). Samples were analyzed in both positive and negative ion modes with a resolution of R_m/z=200_ = 280,000 for MS and R_m/z=200_ = 17,500 for MSMS experiments, in a single acquisition. MSMS was triggered by an inclusion list encompassing corresponding MS mass ranges scanned in 1 Da increments^73^. Both MS and MSMS data were combined to monitor CE, DAG and TAG ions as ammonium adducts ([M + NH_4_]^+^); PC, PC O-, as acetate adducts ([M + CH_3_COO]^-^); and CL, PA, PE, PE O-, PG, PI and PS as deprotonated anions ([M-H]^-^; [M-2H]^-^ in case of CL). MS only was used to monitor LPA, LPE, LPE O-, LPI, and LPS as deprotonated anions ([M-H]^-^); Cer, HexCer, SM, LPC, and LPC O- as acetate adducts ([M + CH_3_COO]^-^) and cholesterol as ammonium adduct([M + NH_4_]^+^) of an acetylated derivative^76^. An overview of MS modes is shown in Suppl. Table S03. Lipid species identified in MS mode only should be considered putative annotations according to guidelines of the Lipidomics-Standards-Initiative (https://lipidomics-standards-initiative.org/guidelines/lipid-species-identification/direct-infusion-ms-esi#).

### Post-processing

Spectra were analyzed with in-house developed lipid identification software based on LipidXplorer^77,78^. Lipids were identified from the high-resolution spectra using LipotypeXplorer software, which uses custom scripts for lipid species identification, as described earlier^72,79^. For each lipid class, a separate software script describing the sum formula constraints in the MS1 and MS2- spectral space was custom-written according to the general building-block rules of lipid identification^80–83^. Constraints for carbon numbers and double bonds of fatty acid chains or long-chain bases were as follows: 10–24 carbon atoms and 0–6 double bonds in the fatty acid moiety, and 16–20 carbon atoms and 0–2 double bonds in the long-chain base moiety.

TAGs are quantified as species (e.g. TAG 48:0;0). Fatty acid amounts within TAG species were calculated based on intensities of neutral losses of fatty acid fragments. Data post-processing and normalization were performed using an in-house developed data management system. Only lipid identifications with a signal-to-noise ratio > 5, and a signal intensity fivefold higher than in corresponding blank samples were considered for further data analysis.

Using 8 reference samples per 96-well plate batch, lipid amounts were corrected for batch variations and for analytical drift if the p-value of the slope was below 0.05 with an R^2^ greater than 0.75 and the relative drift was above 5%. The median coefficient of subspecies variation as accessed by reference samples was 19.17% and 11.2% for the two runs of measurement and using only lipids present in 80% of the samples. An assessment of method performance including a limit of detection (LOD), a limit of quantification (LOQ), dynamic range, and a method precision of various lipid classes has recently been published^84^.

### Data analysis

Data were analyzed with R version 4.0.3^85^ using tidyverse packages version^86^ 1.3.0. We standardized lipid amounts to the total lipid amount (molar fraction [mol%]).

For principal component analysis (PCA) we used all lipids occurring in every replicate of at least one cell line. The dataset was imputed by the caret::preProcess() function using the median impute method^87^ (Fig. 2). The PCA was calculated with the pcaMethods::pca() function^88^ using singular value decomposition and all values were centered and scaled to unit variance.

The data we provide contains 2095 lipid species in 24 lipid classes. In this dataset lipid measurements were included, for which at least 2 valid measurements in any cell line were available (Suppl. Table SO4). The frequency histogram of the number of samples a lipid species has been detected in is shown in Suppl. Table S03.

For comparisons of two clones, only lipid species were used which had at least 2 valid measurements in each of the two cell lines compared. To avoid the loss of knockout-specific lipids, this procedure was applied to each comparison separately, resulting in varying numbers of lipids for each comparison. Missing data were omitted from the test. Lipid species were compared with a two-sided t-test with unequal variance (stats::t.test()) and results were corrected for multiple testing using the stats::p.adjust() function with the method of Benjamini & Hochberg^89^.

For heatmaps, we used the same data as in the PCA analysis, with all lipids occurring in every replicate of at least one cell line. The dataset was imputed by the caret::preProcess() function using the median impute method^88^. The PCA was calculated with the pcaMethods::heatmap3() function with the scale attribute set to “column”^90^.

## Supplementary Information

Supplementary Information 1.

Supplementary Information 2.

Supplementary Information 3.

Supplementary Information 4.

## Data Availability

Data were uploaded to FigShare.com: https://doi.org/10.6084/m9.figshare.14775126. Lipid identifiers of the SwissLipids database (http://www.swisslipids.org) are used. Sequences generated during and analyzed during the current study are available at Genbank (https://www.ncbi.nlm.nih.gov/genbank/). GenBank accession numbers are provided in Suppl. Tables S01 and Table S02.
