# Supplementary material for: A set of gene knockouts as a resource for global lipidomic changes
Source: Sci Rep. 2022 Jun 22;12:10533. doi: 10.1038/s41598-022-14690-0 (PMC9218125; doi:10.1038/s41598-022-14690-0)
Supplement: Supplementary file 1 — Supplementary Information 1. [file 41598_2022_14690_MOESM1_ESM.pdf]

A set of gene knockouts as a resource for global lipidomic changes
Supplemental material

Aleksandra Spiegel[1], Chris Lauber[2], Mandy Bachmann[1], Anne-Kristin Heninger[1], Christian Klose[2], Kai Simons[2], Mihail Sarov[1], Mathias J. Gerl[2*]

[1] Max Planck Institute of Molecular Cell Biology and Genetics, Pfotenhauerstrasse 108, 01307 Dresden, Germany

[2] Lipotype GmbH, Tatzberg 47, 01307 Dresden

[*] Correspondence: gerl@lipotype.com

Contents

Figures 2

Tables 9

Gel originals 18

Figures

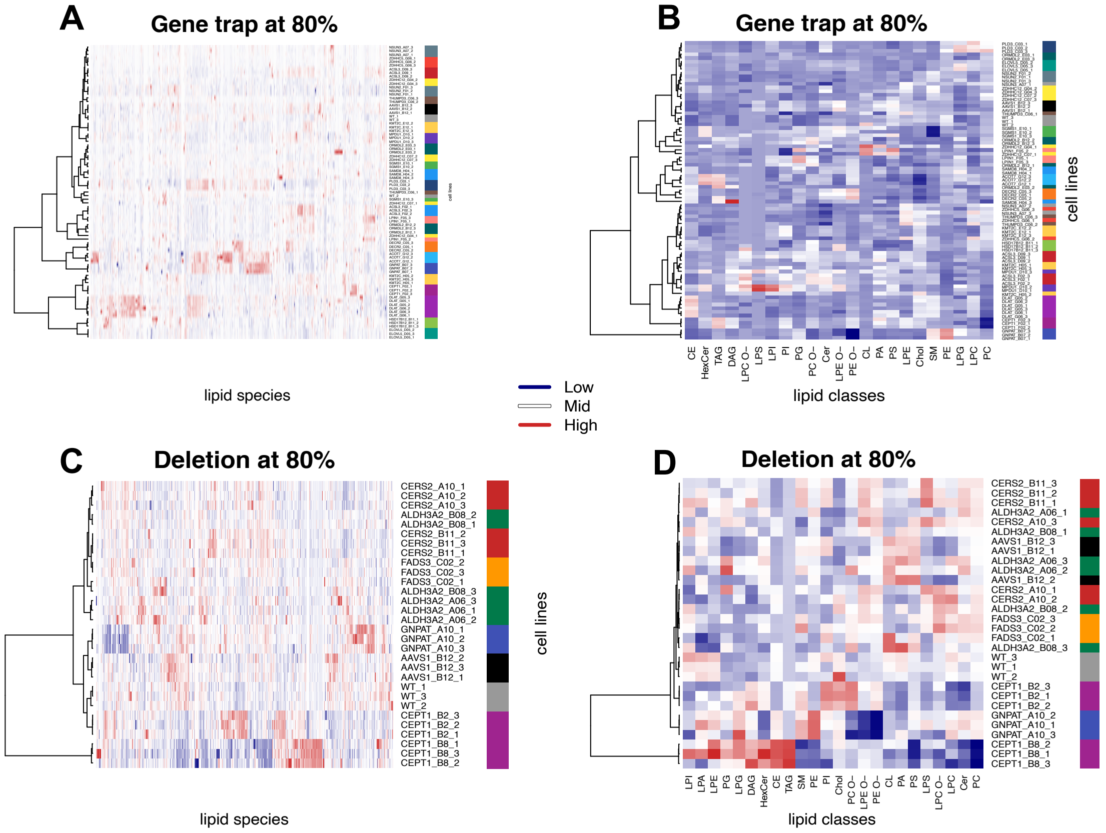

Figure S01: Heatmap of lipid species and classes: **(A)** Heatmap and clustering of all samples in the **gene trap dataset** by using all lipids occurring in every replicate of at least one cell line (n = 1449). Only samples that were generated at 80% confluency with the gene trap method are used. Samples are colored by gene targeted. Note that some genes are represented with one clone (a total of 3 replicates), while other genes are represented by two clones (a total of 6 replicates). **(B)** Same as (A) but only using lipid classes as input. **(C)** Heatmap and clustering of all samples in the **deletion dataset** by using all lipids occurring in every replicate of at least one cell line (n = 1590).**(D)** Same as (C) but only using lipid classes as input.

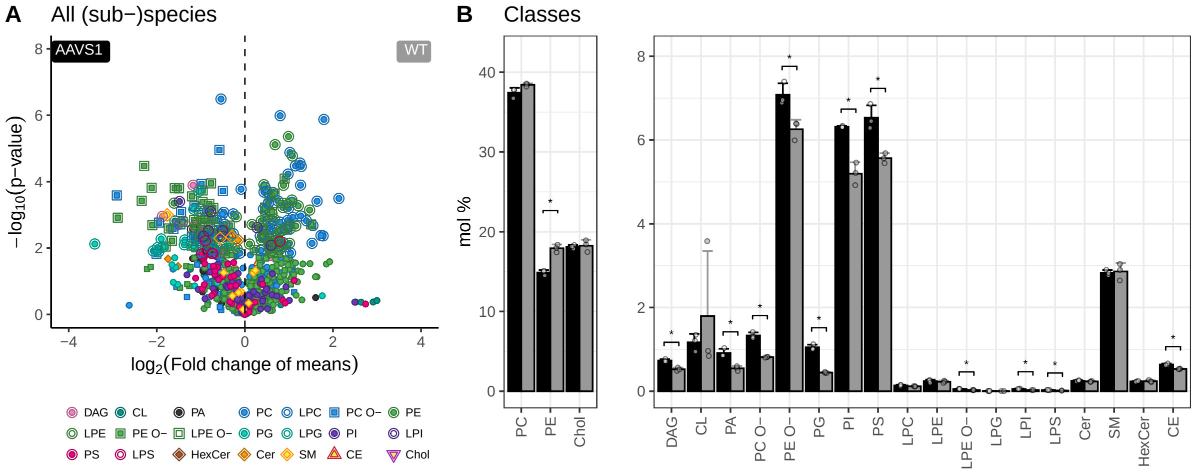

Figure S02: WT vs. AAVS1 control: **(A) Volcano plot**: Lipid species of the WT KO cell line (n = 3) and the AAVS1 control (n = 3) were compared. P-values of t-tests without correction for multiple testing are displayed on the y-axis, fold changes of means are shown on the x-axis. Points with additional outlines indicate lipids significant after correction for multiple testing (Benjamini–Hochberg, 227 lipid species or 30.5% of all species). Shapes and colors of points indicate the lipid class of the species. **(B) Lipid classes**: Values for individual samples are shown by points and means are indicated by bar plots. Error bars correspond to standard deviations. p-values have been adjusted for the total number of classes (n = 21) and are encoded as follows: * for $q < 0.05$, ** for $q < 0.01$, *** for $q < 0.01$, **** for $q < 0.0001$. Only lipid species were used, which had at least 2 valid measurements in each of the two cell lines (n = 744).

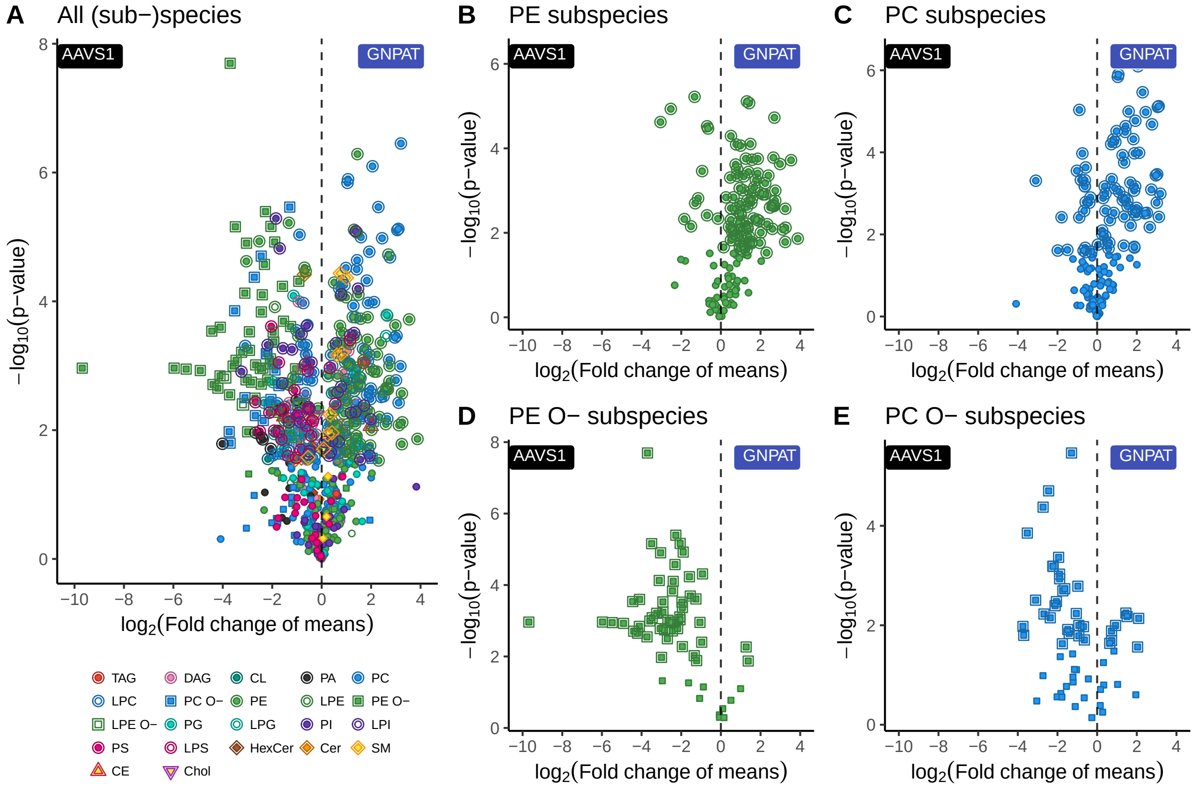

Figure S03: GNPAT knockout: The GNPAT clone B07 is shown, which was created by the gene trap method. **(A) Volcano plot**: Lipid species of the GNPAT KO cell line (n = 3) and the AAVS1 control (n = 3) were compared. P-values of t-tests without correction for multiple testing are displayed on the y-axis, fold changes of means are shown on the x-axis. Points with additional outlines indicate lipids significant after correction for multiple testing (Benjamini–Hochberg, 401 lipid species or 60.1% of all species). Shapes and colors of points indicate the lipid class of the species. **(B) PE sub-species**: Subset of (A). Of a total of 149 PE subspecies, 97 were significant after correcting for multiple testing, with 12 showing elevated levels in the AAVS1 control and 85 showing elevated levels in GNPAT. **(C) PC subspecies**: Subset of (A). Of a total of 153 PC subspecies, 84 were significant after correcting for multiple testing, with 21 showing elevated levels in the AAVS1 control and 63 showing elevated levels in GNPAT. **(D) PE O- subspecies**: Subset of (A). Of a total of 59 PE O- subspecies, 49 were significant after correcting for multiple testing, with 47 showing elevated levels in the AAVS1 control and 2 showing elevated levels in GNPAT. **(E) PC O- subspecies**: Subset of (A). Of a total of 59 PC O- subspecies, 35 were significant after correcting for multiple testing, with 27 showing elevated levels in the AAVS1 control and 8 showing elevated levels in GNPAT. **(A-E)** Only lipid species were used, which had at least 2 valid measurements in each of the two cell lines (n = 667).

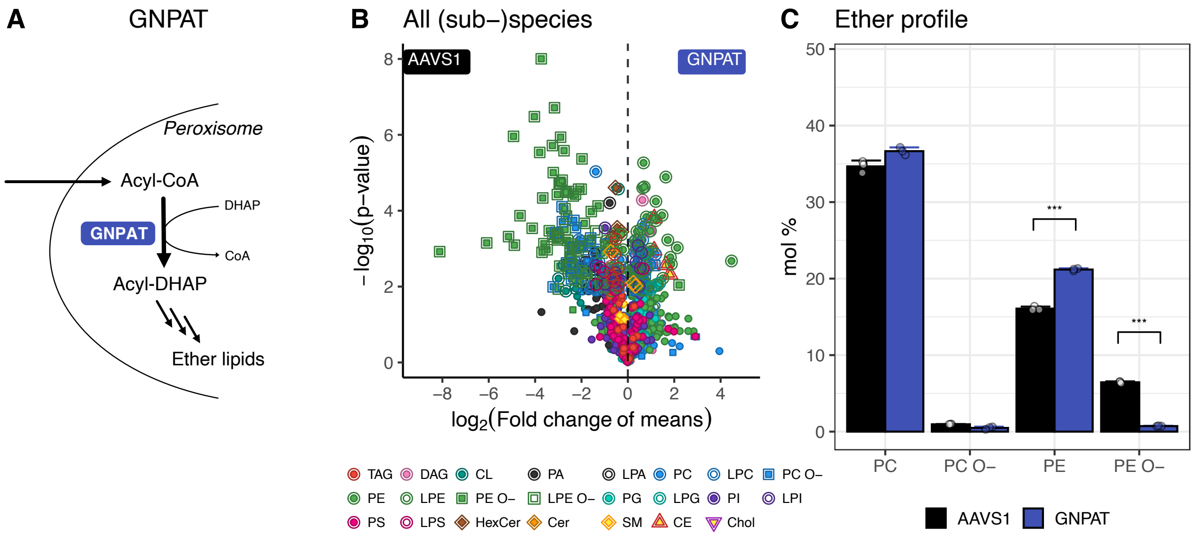

Figure S04: GNPAT knockout control: **(A)** Dihydroxyacetone phosphate acyltransferase (GNPAT) catalyzes the esterification of dihydroxyacetone phosphate (DHAP) with an acyl-CoA, which is the beginning of the ether lipid biosynthesis in peroxisomes. Redrawn from (Zhu et al. 2019) **(B) Volcano plot**: Lipid species of the GNPAT KO cell line (n = 3) and the AAVS1 control (n = 3) were compared. P-values of t-tests without correction for multiple testing are displayed on the y-axis, fold changes of means are shown on the x-axis. Points with additional outlines indicate lipids significant after correction for multiple testing (Benjamini–Hochberg, 262 lipid species or 26.6% of all species). Shapes and colors of points indicate the lipid class of the species. **(C) PC and PE lipid classes**: Diacyl and ether-linked lipid classes are shown. Values for individual samples are shown by points and means are indicated by bar plots. Error bars correspond to standard deviations. p-values have been adjusted for the total number of classes (n = 23) and are represented by stars. **(A-B)** Only lipid species were used, which had at least 2 valid measurements in each of the two cell lines (n = 984). The GNPAT KO clone A10 is displayed, which was created by the deletion method.

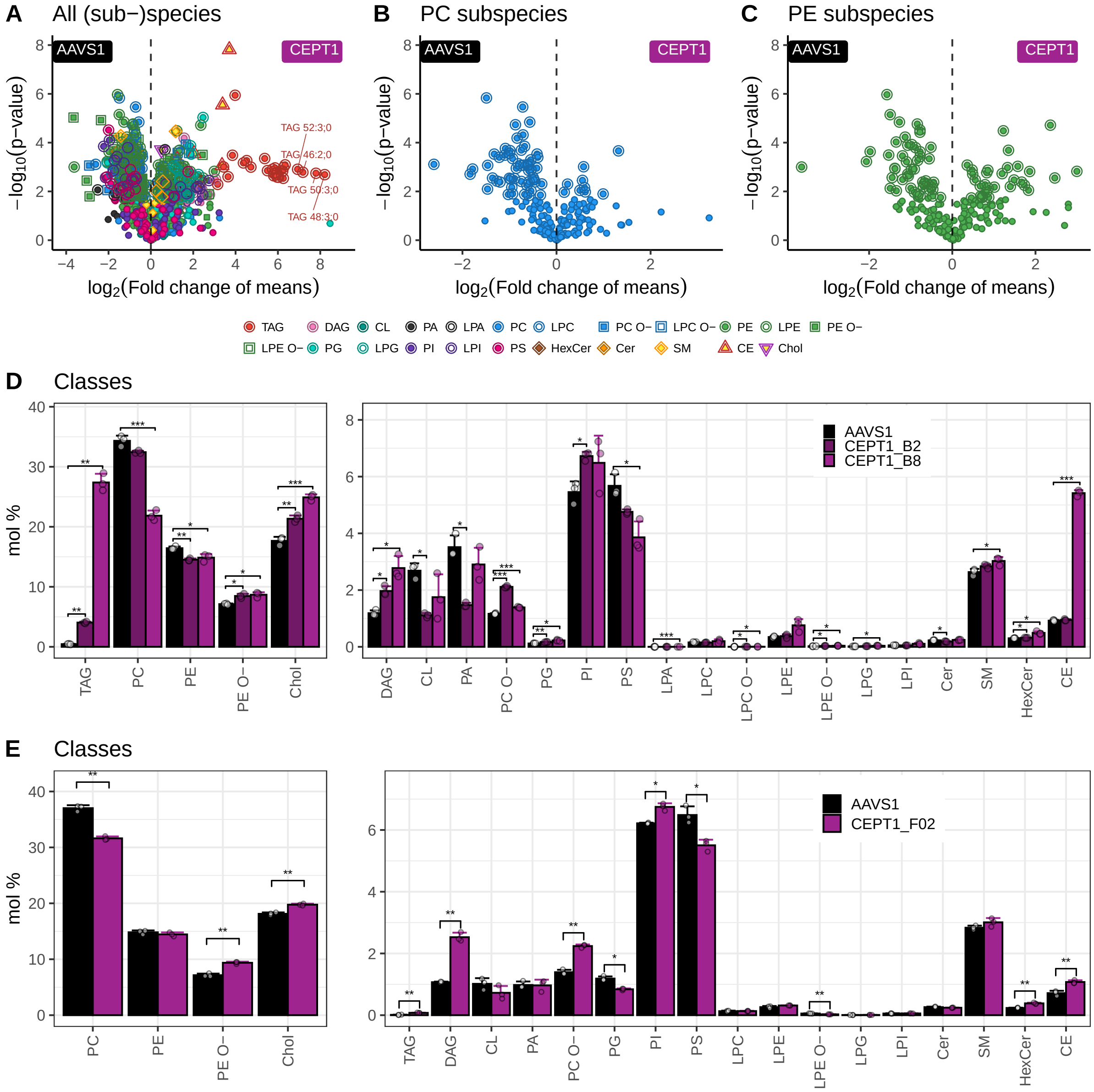

Figure S05: CEPT1 knockout: **(A) Volcano plot**: Lipid species of the CEPT1 KO cell line "B8" clone (n = 3) and the AAVS1 control (n = 3) were compared. P-values of t-tests without correction for multiple testing are displayed on the y-axis, fold changes of means are shown on the x-axis. Points with additional outlines indicate lipids significant after correction for multiple testing (Benjamini–Hochberg, 394 lipid species or 37.5% of all species). TAG species with the highest fold change are highlighted. **(B) PC subspecies**: Subset of (A). Of a total of 191 PC subspecies, 62 were significant after correcting for multiple testing, with 53 showing elevated levels in the AAVS1 control and 9 showing elevated levels in CEPT1. **(C) PE subspecies**: Subset of (A). Of a total of 202 PE subspecies, 77 were significant after correcting for multiple testing, with 47 showing elevated levels in the AAVS1 control and 30 showing elevated levels in CEPT1. **(D) Lipid classes**: Values for individual samples are shown by points and means are indicated by bar plots. Error bars correspond to standard deviations. p-values have been adjusted for the total number of classes (n = 23) and are encoded as follows: * for $q < 0.05$, ** for $q < 0.01$, *** for $q < 0.01$,**** for $q < 0.0001$. **(A-D)** As fold changes in TAG were so dominant, lipid species were standardized to total lipid amount without TAG. Only lipid species were used, which had at least 2 valid measurements in each of the two cell lines (n = 1052). The CEPT1 KO clones B2 and B8 are displayed, which were created by the deletion method.**(E) Lipid classes**: Same as (D). The CEPT1 KO clone F02 is displayed, which was created by the gene trap method. Only lipid species were used, which had at least 2 valid measurements in each of the two cell lines (n = 740).

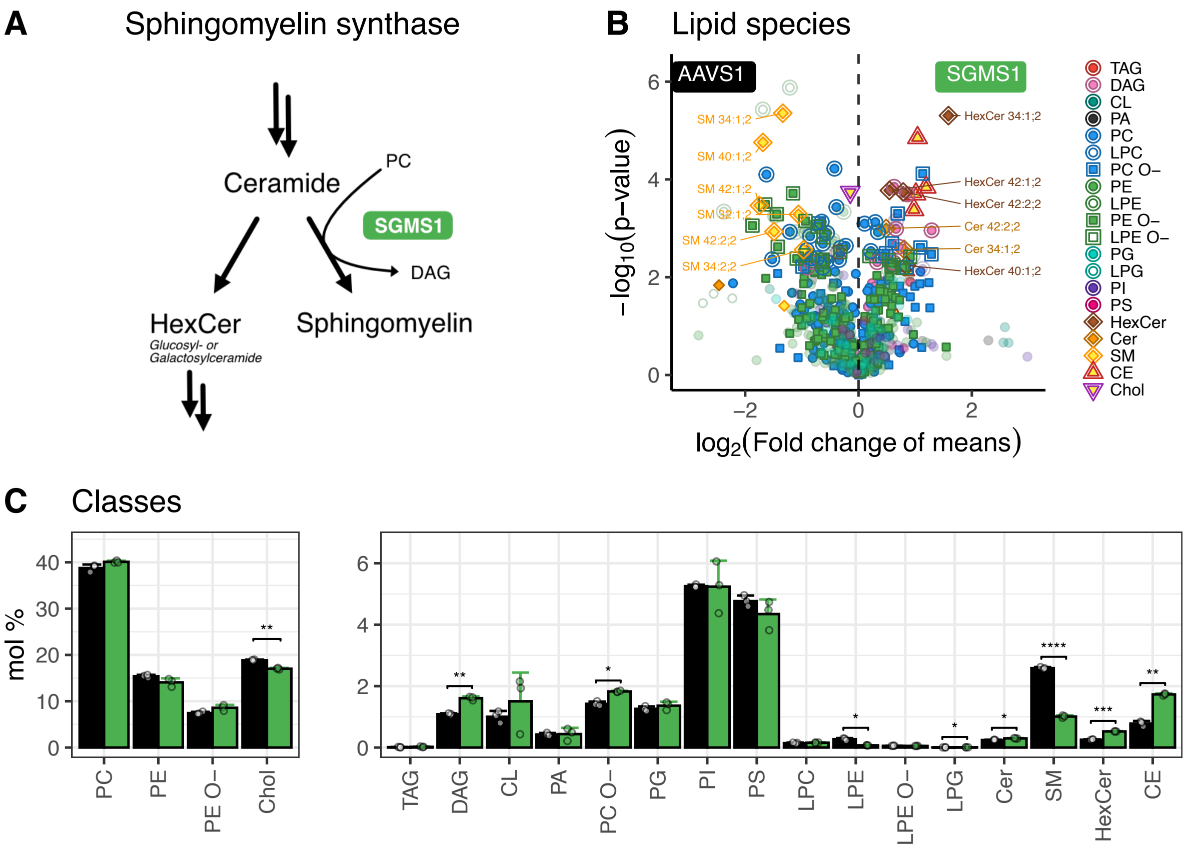

Figure S06: SGMS1 knockout: **(A)** Sphingomyelin synthase transfers the phosphocholine head group of phosphatidylcholine (PC) on to ceramide (CER) to form sphingomyelin (SM) and diacyl-glycerol (DAG). **(B) Volcano plot**: Lipid species of the SGMS1 KO cell line (n = 3) and the AAVS1 control (n = 3) were compared. P-values of t-tests without correction for multiple testing are displayed on the y-axis, fold changes of means are shown on the x-axis. Points with additional outlines indicate lipids significant after correction for multiple testing (Benjamini–Hochberg, 97 lipid species or 14.3% of all species). Shapes and colors of points indicate the lipid class of the species. Sphingolipid species are labeled by their species names. **(C) Lipid classes**: Values for individual samples are shown by points and means are indicated by bar plots. Error bars correspond to standard deviations. p-values have been adjusted for the total number of classes (n = 20) and are encoded as follows: * for q < 0.05, ** for q < 0.01, *** for q < 0.01,**** for q < 0.0001. Only lipid species were used, which had at least 2 valid measurements in each of the two cell lines (n = 677). The SGMS1 KO clone E10 is displayed, which was created by the gene trap method.

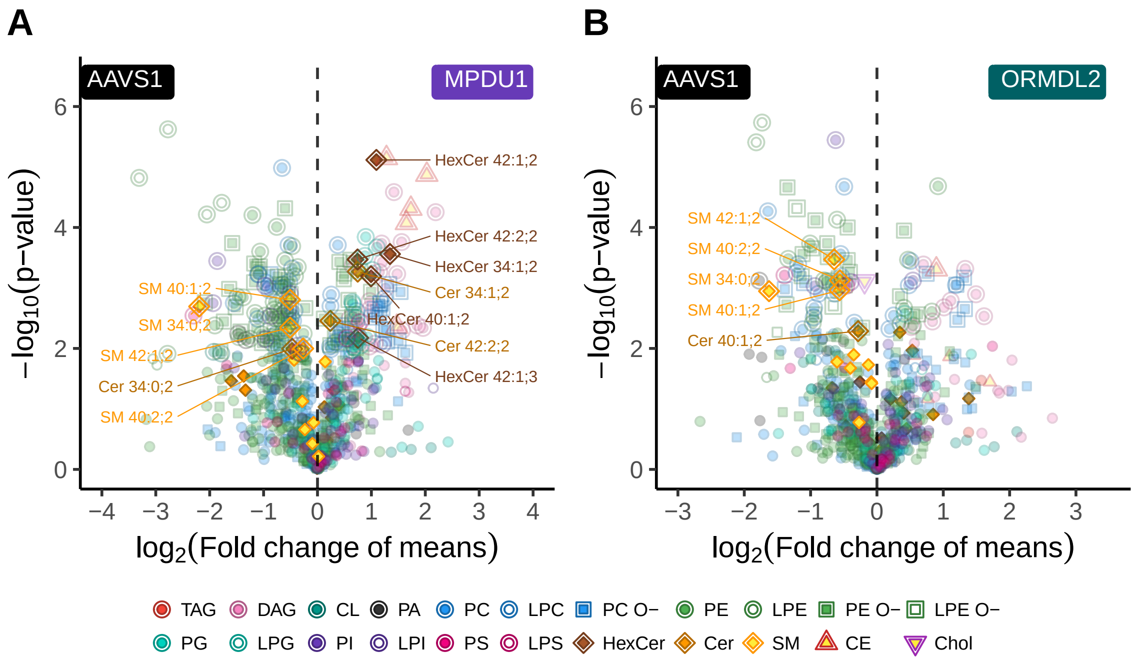

Figure S07: MPDU1 KO and ORMDL2 KO: **(A) MPDU1 KO Volcano plot**: Lipid species of the MPDU1 KO cell line (n = 3) and the AAVS1 control (n = 3) were compared. P-values of t-tests without correction for multiple testing are displayed on the y-axis, fold changes of means are shown on the x-axis. Points with additional outlines indicate lipids significant after correction for multiple testing (Benjamini–Hochberg, 197 lipid species or 25.2% of all species). Shapes and colors of points indicate the lipid class of the species. Sphingolipid species are labeled by their species names. Only lipid species were used, which had at least 2 valid measurements in each of the two cell lines (n = 783). The MPDU1 KO clone D10 is displayed, which was created by the gene trap method. **(B) ORMDL2 KO Volcano plot**: Lipid species of the ORMDL2 KO cell lines (n = 6) and the AAVS1 control (n = 3) were compared. P-values of t-tests without correction for multiple testing are displayed on the y-axis, fold changes of means are shown on the x-axis. Points with additional outlines indicate lipids significant after correction for multiple testing (Benjamini–Hochberg, 84 lipid species or 10.6% of all species). Sphingolipid species are labeled by their species names. Only lipid species were used, which had at least 2 valid measurements in each of the two cell lines (n = 795). The ORMDL2 KO clones B12, E03 are displayed, which were created by the gene trap method.

Tables

Table S01 Gene Trap approach — Gene Targeting Designs: Genes knocked out by in-frame insertion of the stop cassette (gene trap). See below for the table and for Gel originals.

Table S02 Deletion approach — Gene Targeting Designs: Genes knocked out by frameshift/critical exon deletion or by truncation of the functional domain. See below.

Table S03 Multiple tables on lipid identification and quality control. **Tab "SwissLipids IDs"**: SwissLipids Identifiers for lipids in this study (Table S04). **Tab "MS modes"**: List of analyzed lipid classes and detection mode. MSMS mode (i.e. the fragmentation of the lipid molecules) delivers additional confidence in lipid identification and additional structural information. **Tab "Detection frequency histogram"**: A frequency histogram of the number of cell lines a lipid species has been detected in. **Tab "Coefficients of Variation"**: Coefficients of Variation (CV) are provided for each lipid species and each gene knockout across replicates, clones, and methods. A separate CV is calculated for each growth state. In addition, CVs are calculated across all samples for the different growth states ("across all samples"). Summary statistics of CV values are provided across all lipid species with the minimal value (min), first quartile (Q1), median, third quartile (Q3), and the maximal value (max) for each knockout or group. Further, summary statistics of all available CVs calculated within each knockout are provided ("within each sample"). Data were uploaded to FigShare.com: DOI: 10.6084/m9.figshare.15019590

Table S04 Lipidomic measurements as molar fraction (mol%). Also containing: the gene knocked out (gene), the respective clone, the growth state (20% or 80% confluency), the method of knockout (gene trap or deletion), the genotype, and the total amount of lipid found in the measurement (total pmol). Lipid measurements were included in the dataset, if there were at least 2 valid measurements in any cell line. The fatty acid profile (in species percent) is also provided for each TAG species. Data were uploaded to FigShare.com: DOI: 10.6084/m9.figshare.14775126.

Table S05 Results of statistical tests. **Tab "Gene names"**: Uniprot IDs and Protein names of the genes knocked out in this study. **Tab "Overview comparisons"**: Overview of all comparisons displayed in the file, including the tab names, IDs used, number of replicates, and number of lipids compared. **Tabs on statistical tests** contain 4 sets of comparisons: lipid class, fatty acid (FA), fatty acids with a lipid class (class FA), and (sub-)species, with mean value, standard deviation, number of replicates of the feature (n), fold change of means, p-value and the p-value corrected for multiple testing based on the set (BH). Data were uploaded to FigShare.com: DOI: 10.6084/m9.figshare.16644928

Table S01 Genes knocked by in-frame insertion of the stop cassette (gene trap).

Gene	GenBank accession	Type	1) Homology arm sequence (LEFT HA, right HA; 5' → 3')<br>2) Sanger sequencing of mut allele after NHEJ (where applicable)	crRNA sequence (5' → 3')	Genotyping primers (5' → 3')	Genotyping PCR
ACOT7	Targeting construct: OM778728	KI/KI	1) AAGGTTCTCTTTCCTCCGGGAGCCATTGAGCCCTTTGGGGACCTTCTCCCCTCCTTGGATTGTCACTGTTGCACAAATGGCATCCTCTCCAGAGGCCTCATCCTGCTTCTCCTCTGCCCTAGGATCATGCGGCCAGATGATGCCAACGTGgccggcaatgtccacgggggaccatcctgaagatgatcgaggaggcaggcgccatcatcagcacccggcattgcaacagccagaacggggtaagggtctgggccccatacctctgggccctggaggccgcaccagtgtgagttcaggac	GTGGACATTGCCGGCCACGT	Flanking (F):<br>Fwd: CATGGAAACAGCATCTCAGG<br>Rev: ACGTCCCCGCATGTTAGAAG (T2A-rev)<br><br>Spanning (S):<br>Fwd: CATGGAAACAGCATCTCAGG<br>Rev: CCACTTCTCCCACAGTTCC	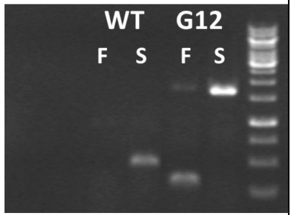
ACSL3	Targeting construct: OM778727	KI/KI<br>KI/KI	1) CTTTATACTATTTTAACATACATTCCGTTTTATTTTTTCTCCGAGTCAAGACAAGAAAAATCAAACCGAATTAAAGCAAAGCCTGTAAATTCAAAACCTGATTCTGCATACAGATCTGTTAATAGTTTGGATGGTTTGGCTTCAGTATTAtaccctggatgtgatactttagataaagtttttacatatgcaaaaaacaaatttaagaacaaaagactcttgggaacacgtgaagttttaaatgaggaagatgaagtacaaccaaatggaaaaatttttaaaaaggtaagatgatctttc	TTGGCTTCAGTATTATACCC	Flanking (F):<br>Fwd: GGTAGCTGCTGCCTGATAG<br>Rev: ACGTCCCCGCATGTTAGAAG (T2A-rev)<br><br>Spanning (S):<br>Fwd: GGTAGCTGCTGCCTGATAG<br>Rev: TCTAGCCCCCTTCCTACAC	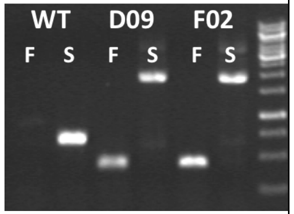
CEPT1	Targeting construct: OM778726	KI/KI	1) AGATGTAAATAGTGGCCAACAAGTGTATTTTTTAATTTTTATTTTATTTTATTTTTTAGGTAAGCACCAGCCACAAAAACCTACAAAAGAAGGGAAATTACTGTCTTTAAATATTAAAAAAAAACAAGATCCATGAGTGGGCATCGATCAacaaggaaaagatgtggagattctcacccggagtccccagtgggcttcgggcatatgagtactacaggatgtgtattaaataaattgtttcagttaccaacaccaccattgtcaagacaccaactaaagcggctagaagaacacagatat	TGAGTGGGCATCGATCAACA	Flanking (F):<br>Fwd: GGTCCCTCCTTTTCTGGTC<br>Rev: ACGTCCCCGCATGTTAGAAG (T2A-rev)<br><br>Spanning (S):<br>Fwd: GGTCCCTCCTTTTCTGGTC<br>Rev: ATTTGGGGCAATCCAGGAGG	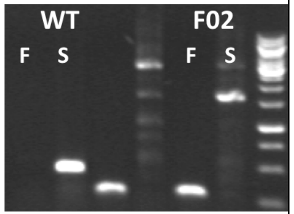
DECR2	Targeting construct: OM778725	KI/KI	1) CTGTGCCCTCTGCCAGCTGGGCCTTCAGCCTGGACTGGTACTTCCGCCTGAGAGACTCCTGCTCAGGAGCTGGGCAGATGCCCCGGGAGTGCCCCTCGCCCACACTGCCCTCACACCTGCTTCTGGTTTTGCAGGCACGGCTGCCATACGgtgattgccagtaggagcctgccgcgagtgctgacggtgagagggcctctcccatggtcccctgttcgggtggctgtgggggggctggggctgggcctgggccaggagagtccagaggctatggggatgttggcaccaaaactttttttt	TCCTACTGGCAATCACCGTA	Flanking (F):<br>Fwd: AGAGTACCTCAGTCCTGTTC<br>Rev: ACGTCCCCGCATGTTAGAAG (T2A-rev)<br><br>Spanning (S):<br>Fwd: AGAGTACCTCAGTCCTGTTC<br>Rev: AGATCGCACCACCACACTTC	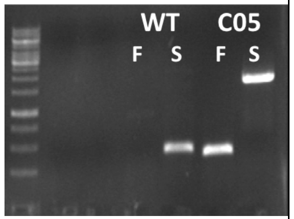
DLAT	Targeting construct: OM778724	KI/KI<br>KI/KI	1) CGACAACGTGCGGCTGTGCGGTGTGGCTGACGGCAACGCCGCTGCTCTTGGAGAGGTCACTCCGGAGACGGCGTTGGTTTTGGGGTGTGGGGGGTTGGTGGCACTATGTGGCGCGTCTGTGCGCGACGGGCTCAGAATGTAGCCCCATGGgcgggactcgaggctcggtggacggccttgcaggaggtacccggaactccacgagtgacctcgcgatctggcccggctcccgctcgtcgcaacagcgtgactacagggtatggcggggtccgggcactgtgcggctggacccccagttct	GAGCCTCGAGTCCCGCCCAT	Flanking (F):<br>Fwd: TTTCGGATGCCTCCCCTAG<br>Rev: ACGTCCCCGCATGTTAGAAG (T2A-rev)<br><br>Spanning (S):<br>Fwd: TTTCGGATGCCTCCCCTAG<br>Rev: TCTTGCAGGCATCCAGTCAG	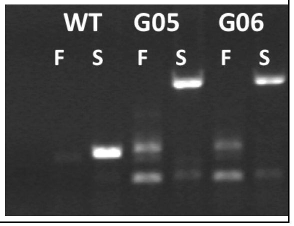

Gene	GenBank accession	Type	1) Homology arm sequence (LEFT HA, right HA; 5' → 3')<br>2) Sanger sequencing of mut allele after NHEJ (where applicable)	crRNA sequence (5' → 3')	Genotyping primers (5' → 3')	Genotyping PCR
ELOVL5	Targeting construct: OM778723	KI/KI	1) ATTTTTTTTAATTTTTCAGATACTAGAGTAAAAGGATGGTTTCTTCTGGACAATTATATACCCACA TTTATCTGCTCTGTCATATATTTACTAATTGTATGGCTGGGACCAAAATACATGAGGAATAAACA GCCATTCTCTTGCCGGGGGattttagtggtgtataaccttggactcacactgctgtctctgtatatgttctgtgaggtaa gtcaaaggttaacacagtcttcctttcacataaatgcactcatattgctgtcctaaattgtattcctaaatgaggaaaggtggcc gctt	TATACACCACTAAAATCCCC	Flanking (F):<br>Fwd: AGGTGACTTTTATATCATCATGG<br>Rev: ACGTCCCCGCATGTTAGAAG (T2A-rev)<br><br>Spanning (S):<br>Fwd: AGGTGACTTTTATATCATCATGG<br>Rev: TTGCTTGCCCAGTTGTCTCT	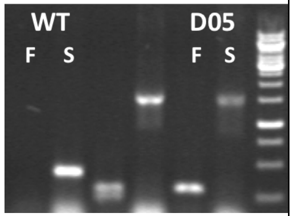
GNPAT	Targeting construct: OM778722	KI/KI	1) TTTGAGTTATAGAAGATTAATCCATTTGGAACTAGTCATACTGGTTTGAGTAAAGCTTTCTGACT TTTCTGTTATTGTTGTTAGAAATTATTCCTGCTTTTCCTTTCCTTTTAGCTTTCCAAGGAATCCCTT CAATCTGTGGATGTCCTCcgagaggaagtgagtgagatcttagatgaaatgagtcacaaactgcgtcttggagccat tcggttttgtgccttcaccctgagcaaagtatttaaacaaattttctcgaaggtgtgtgtaaatgaagaaggtattcagaaagt gagtatt	ATCTGTGGATGTCCTCCGAG	Flanking (F):<br>Fwd: TGACTGTGTTGGGAAGTTGTC<br>Rev: ACGTCCCCGCATGTTAGAAG (T2A-rev)<br><br>Spanning (S):<br>Fwd: TGACTGTGTTGGGAAGTTGTC<br>Rev: ATGCACATCCTCTTCTGCCT	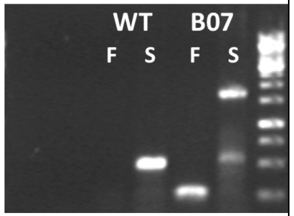
HSD17B12	Targeting construct: OM778721	KI/KI	1) GCTGGTATTGTTCTGTCTTTTACATCAAAGTGTCACACTGGTCCTTTTGTATGTAATTGGTGGGT CCTAACTATGACCAATTCCTCAATGTAATTCTTAGACTAAACTATTGCTTTTTTCCCTTTCCCAGTT GTCACAGGTAGTACTGATggaattggaaaatcatatgcagaagaggtaggtgattttcaagatctttccttttaatata aaaaataagaataaatatgggatgatttcttatagtagttttgatttttgaagtaaaaaacacaaatataatgttgcatggtac aatag	ACAGGTAGTACTGATGGAAT	Flanking (F):<br>Fwd: CCACCAGCAATGTGTGAGAG<br>Rev: ACGTCCCCGCATGTTAGAAG (T2A-rev)<br><br>Spanning (S):<br>Fwd: CCACCAGCAATGTGTGAGAG<br>Rev: CCTGCTTTGTCCAGTGGTC	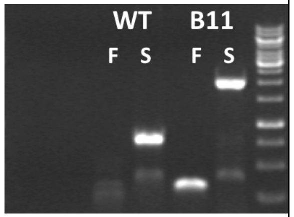
KMT2C	Targeting construct: OM778720<br><br>Mut allele (clone E12): OM778740	KI/mut<br>KI/KI	1) AAACACAAAAATGACTCTTTGTTTTCTCTTGTTTACTGTTAAGCAGAAATCAAAGAACAATCTGC AGAAGAGGATGCTGAAGCAGAAGTGGATAACAGCAAACAGCTAATTCCAACTCTTCAGCGATC TGTGTCTGAGGAATCGGCAAACtccctggtctctgttggtgtagaagccaaaatcaggttagagaatgaatgcagc tggattggatattagtgacaggaataaagcagtgaaatgactatagagaaaattcaaggagtatgagtttaggatgaaaatc tgggtgggatttta<br><br>2) mut allele sequence (clone E12)<br>TGTTAAGCAGAAATCAAAGAACAATCTGCAGAAGAGGATGCTGAAGCAGAAGTGGATAACAG CAAACAGCTAATTCCAACTCTTCAGCGATCTGTGTCTG[deletion:20bp]GTCTCTGTTGGTGTA GAAGCCAAAATCAGGTTAGAGAATGAATGCAGCTGGATTGGATATTAGTGACAGGAATAAAGC AGTGAAATGACTATAGAGAAAA	TGAGGAATCGGCAAACTCCC	Flanking (F):<br>Fwd: GCTCAAGGGATTTGCTTGC<br>Rev: ACGTCCCCGCATGTTAGAAG (T2A-rev)<br><br>Spanning (S):<br>Fwd: GCTCAAGGGATTTGCTTGC<br>Rev: CTGTACTGGCCTGAGGTGA	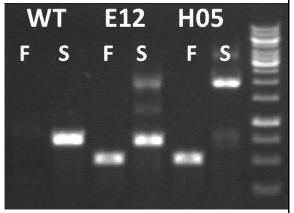
LPIN1	Targeting construct: OM778730<br><br>Mut allele (clone F05): OM778741	KI/mut	1) GTGTCTGTGTGTGTTTTTTTTTGTCTGTTTTCCAGGTGCAGACCATGAATtacgtggggcagttagccgg ccaggtgtttgtcaccgtgaaggagctcta<br><br>2) mut allele sequence (clone F05)<br>TGTGTAATCCACGTTTTTGAAATGGTGAGGAGTTCATTTTGATTGGCTCTTCCTTGGATTAATTG TGTGTCTGTGTGTGTTTTTTT[deletion:2bp]GTCTGTTTTCCAGG[deletion:20bp]GCAGTTAG CCGGCCAGGTGTTTGTCACCGTGAAGGAGCTCTACAAGGGGCTGAATCCCGCCACACTCTCAG GGTGCATTGACATCATTGTCATCCGCCAG	GTGCAGACCATGAATTACGT	Flanking (F):<br>Fwd: TGGGCTATGGGGGTGGATA<br>Rev: ACGTCCCCGCATGTTAGAAG (T2A-rev)<br><br>Spanning (S):<br>Fwd: TGGGCTATGGGGGTGGATA<br>Rev: CACCACTTTCTCTCGGGAG	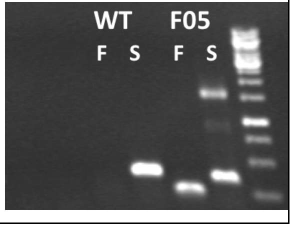

Gene	GenBank accession	Type	1) Homology arm sequence (LEFT HA, right HA; 5' → 3')<br>2) Sanger sequencing of mut allele after NHEJ (where applicable)	crRNA sequence (5' → 3')	Genotyping primers (5' → 3')	Genotyping PCR
MPDU1	Targeting construct: OM778719	KI/KI	1) GCGGGGCACGGGGCGGGCCAGCTTCGCCGCGGGAAAAGAACGGGAGGCGGAGTGTCCGCAG<br>CGCGCACGCGCAACGAAAGTCAATGGCGGTCTGGAGAGACTGGCGGAAGCTAGCTTTGCAAT<br>ATGGCGGCCGAGGCGGACGGACCGCTTaaacggctgctcgtgccgattcttttacctgagaaatgctacgacca<br>acttttcgttcagtgggacttgcttcacggtgagttttattcagcatccgatccaagtcctactcgagtgaccgtgggcccttagt<br>ccaagccttgatcggcg	GGCACGAGCAGCCGTTTAAG	Flanking (F):<br>Fwd: ACTGGAAGCGTCAACAAAGG<br>Rev: ACGTCCCCGCATGTTAGAAG (T2A-rev)<br><br>Spanning (S):<br>Fwd: ACTGGAAGCGTCAACAAAGG<br>Rev: AATTCCACCCGGTGACTTC	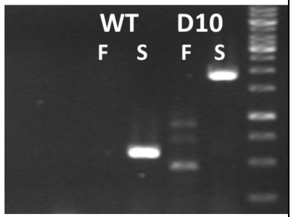
NSUN2	Targeting construct: OM778718	KI/KI	1) TCCTCCTGGGCTTCCCGGACCCCGAGGCCTGCGAGGCGGCGGCGAGGGGGCGGCTCCTTGGG<br>CCTCGGGGTAGACGTCTCTTTCCTTCCTCAGGGCTGGGAAGGAGGCTACCCCGAGATCGTCAA<br>GGAGAACAAGCTGTTCGAGCACTACtaccaggagctcaagatcgtgcccgagggcgagtggggccagttcatgg<br>acgctctcagggagccgctcccggccactttaagaattactggttacaaaaggtagggaggcagtgcctgatttttgggagtt<br>ggggccaggatgctgga	GCTGTTCGAGCACTACTACC	Flanking (F):<br>Fwd: TGCGCTTTGGGCTTATTCCT<br>Rev: ACGTCCCCGCATGTTAGAAG (T2A-rev)<br><br>Spanning (S):<br>Fwd: TGCGCTTTGGGCTTATTCCT<br>Rev: TGTCGAAGAAAACACCGTCG	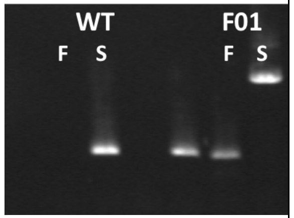
NSUN3	Targeting construct: OM778717<br><br>Mut allele (clone A07): OM778742	KI/mut	1) AGTGAAGTGTAAAACTAGGACCACATTCACCTGATTCGTAATATAAAATTGTATTGGTATGTAT<br>CATATTAATATTCTCTTATTTTCTCTTTTTCTATTTCTAGGGAGATACTAACATCTCCATCATGCTG<br>GCAATATGCTGTCCTGCTTaaccgattcaattatccttttgaactggaaaaggatttacatttgaagggctatcacaca<br>ctctctcagggatctttacccaactatcctaaatcagtgaagtgttaccttagcagaactccgggccgaatcccttcagaaaga<br>caccaa<br><br>2) mut allele sequence (clone A07)<br>TGTATTGGTATGTATCATATTAATATTCTCTTATTTTATCTTTTTCTATTTCTAGGGAGATACTAAC<br>ATCTCCATCATGCTGGCAATATGCTGTCCTGCT[deletion:1bp]AACCGATTCAATTATCCTTTTG<br>AACTGGAAAAGGATTTACATTTGAAGGGCTATCACACACTCTCTCAGGGATCTTTACCCAACTAT<br>CCTAAATCAGTGA	GATAATTGAATCGGTTAAGC	Flanking (F):<br>Fwd: AAAGGGAGTAGCGCCTGGT<br>Rev: ACGTCCCCGCATGTTAGAAG (T2A-rev)<br><br>Spanning (S):<br>Fwd: AAAGGGAGTAGCGCCTGGT<br>Rev: AGGAGCAGCACAGAGATCC	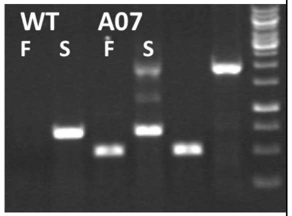
ORMDL2	Targeting construct: OM778716	KI/KI<br>KI/KI	1) TTCAGGGGATTGGGGCTATCTGAGAGACAACTGGGAGATAGCTCAAGAGGGGTTGAGAAAAA<br>ACTGAGCTGGACTCCTGCCTGATCCCCCATTACGGCTAGGATGAATGTGGGGGTGGCACACAG<br>CGAAGTAAACCCCAACACCCGAGTGatgaatagccgaggcatctggctggcctacatcatcttggtaggattgctg<br>catatggttctactcagcatccccttcttcagcattcctgttgtctggaccctgaccaacgtcatccataacctggtgagcacta<br>aaccacgcccttgt	TGCCTCGGCTATTCATCACT	Flanking (F):<br>Fwd: TCTGGGTGTACGTGACCTC<br>Rev: ACGTCCCCGCATGTTAGAAG (T2A-rev)<br><br>Spanning (S):<br>Fwd: TCTGGGTGTACGTGACCTC<br>Rev: CCAGTGTGTCAGTAGCCGA	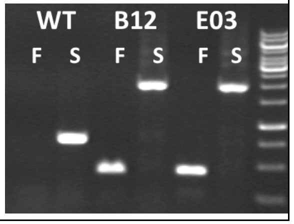

Gene	GenBank accession	Type	1) Homology arm sequence (LEFT HA, right HA; 5’ → 3’)<br>2) Sanger sequencing of mut allele after NHEJ (where applicable)	crRNA sequence (5’ → 3’)	Genotyping primers (5’ → 3’)	Genotyping PCR
PLD3	Targeting construct: OM778715<br><br>Mut allele (clone C03): OM778743	KI/mut	1) CAGGCTGGGCTGCCCCCCTCGGGCTGGCTGACCACCTCCTCCTCCCCACAGAAAGCCCGCTGGG<br>TCCTGCTGGTCCTCATTCTGGCGGTTGTGGGCTTCGGAGCCCTGATGACTCAGCTGTTTCTATGG<br>GAATACGGCGACTTGCATCTCtttgggcccaaccagcgcccagccccctgctatgacccttgcgagtaagtgggggg<br>tgctgcagttggtgggggaggggcctgccagaccaggtacacttaagcacacactaaacagggcctgcactcagccctaccc<br>agcgcttgcgac<br><br>2) mut allele sequence (clone C03)<br>CCTCCCCACAGAAAGCCCGCTGGGTCCTGCTGGTCCTCATTCTGGCGGTTGTGGGCTTCGGAGC<br>CCTGATGACTCAGCTGTTTCTATGGGAATACGGCGA[deletion:28bp]CCAGCCCCCTGCTATGA<br>CCCTTGCGAGTAAGTGGGGG[deletion:1bp]TGCTGCAGTTGGTGGGGGAGGGGCCTGCCAGA<br>CCAGGTACACTTAAGCACACACTAAACAGGG	ACGGCGACTTGCATCTCTTT	Flanking (F):<br>Fwd: ATGAGATTGAGGCGTGGAAG<br>Rev: ACGTCCCCGCATGTTAGAAG (T2A-rev)<br><br>Spanning (S):<br>Fwd: ATGAGATTGAGGCGTGGAAG<br>Rev: AGCCTGCAACCTCCTCAC	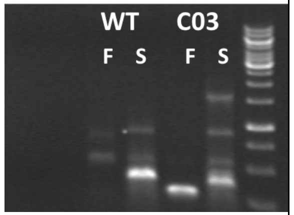
SAMD8	Targeting construct: OM778714	KI/KI	1) GCCTGGCCTGTTTCTGGTGTATTTATGTGTGTTTGTCTTTGAAGAAATTGTTAGGACATACTGAA<br>GCTTTTGTTTTGTTTTCCTGTTTTCTCTCTACAGGCAGCGGAGGAGGAAATGGCAGGTCCTAATC<br>AACTCTGCATTCGCCGCTGGactaccaagcatgtagctgtgtggctgaaggatgaaggctttttttgaatatgtggaca<br>ttttatgcaataagcaccgacttgatggaatcacattgctaacattgactgaatatgatctccggtctcctcctctggaaatcaa<br>agtctta	CTACATGCTTGGTAGTCCAG	Flanking (F):<br>Fwd: TTGACCTCCCAGACTCAGG<br>Rev: ACGTCCCCGCATGTTAGAAG (T2A-rev)<br><br>Spanning (S):<br>Fwd: TTGACCTCCCAGACTCAGG<br>Rev: ATGTCAGGCACTCGCTCATG	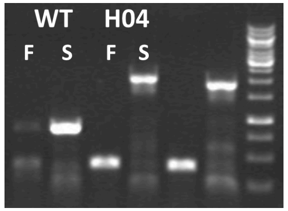
SGMS1	Targeting construct: OM778713<br><br>Mut allele (clone C02): OM778744	KI/mut<br>KI/KI	1) ATCAACCTAACCCAAGAGGATTTCAAAAAACCCCCCTTGTGCCGAGTCTCCTCTGACAATGGGC<br>AGCGGCTCCTGGACATGATAGAAACCCTGAAAATGGAGCACCATTTGGAAGCACACAAGAACG<br>GCCATGCCAATGGGCACCTCAACattggcgtagacatccccacccccgacggcagcttcagcatcaagattaaacc<br>caacgggatgccaaatgggtataggaaagagatgataaagatccccatgccagaactggagcgctctcagtaccccatgga<br>gtggggcaagactttt<br><br>2) mut allele sequence (clone C02)<br>GTCTCCTCTGACAATGGGCAGCGGCTCCTGGACATGATAGAAACCCTGAAAATGGAGCACCAT<br>TTGGAAGCACACAAGAACGGCCATGCCAATGGGCACC[deletion:5bp]ATTGGCGTAGACATCC<br>CCACCCCCGACGGCAGCTTCAGCATCAAGATTAAACCCAACGGGATGCCAAATGGGTATAGGA<br>AAGAGATGATAAAGATCCCCA	GGATGTCTACGCCAATGTTG	Flanking (F):<br>Fwd: GTCGGAACAGTGACTGCTG<br>Rev: ACGTCCCCGCATGTTAGAAG (T2A-rev)<br><br>Spanning (S):<br>Fwd: GTCGGAACAGTGACTGCTG<br>Rev: TTCGTGGACGACCGAGATC	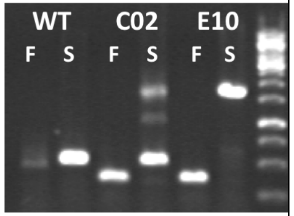
THUMPD3	Targeting construct: OM778712<br><br>Mut allele (clone C06): OM778745	KI/mut	1) GCCCATGTTTTTAATTATAACTGAAATATGAGATGATAATATTTGGGCTTTATTTCTTTTTCTTTT<br>AAAGGACAGTACTGCTTTTAAAGAGACAGTGTTAGGGATCTTGGAAGCACAGCCAACATGTGT<br>GACATTGAAGAAGCCACTAACcaactcctagatgtgaaccttcatgagaaccagaagtctgtacaagtgacagaaa<br>gtgacctcggaagtgaatctgagcttctagtcactattggagccactgtacctactggctttgagcaaacagctgcagatgaa<br>gtcagagagaaa<br><br>2) mut allele sequence (clone C06)<br>TTTCTTTTTCTTTTAAAGGACAGTACTGCTTTTAAAGAGACAGTGTTAGGGATCTTGGAAGCACA<br>GCCAACATGTGTGACATTGAAGAAGCCACTAACTC[insertion:CGGTAGAGCCGAGGGCAGGG<br>GAAGTCTTCTAACATGCGGGGACGTGCTCTATGGCATTGCAGCCCCTG]ACTCCTAGATGTGAA<br>CCTTCATGAGAACCAGAAGTCTGTACAAGTGACAGAAAGTGACCTCGGAAGTGAATCTGAGCT<br>TCTAGTCACTATTGGAGCCACT	CACATCTAGGAGTTGGTTAG	Flanking (F):<br>Fwd: TGTTGCCTTGTTCTTCCTGC<br>Rev: ACGTCCCCGCATGTTAGAAG (T2A-rev)<br><br>Spanning (S):<br>Fwd: TGTTGCCTTGTTCTTCCTGC<br>Rev: CCTGTGCCAGACTTTCCAC	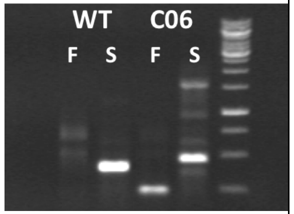

Gene	GenBank accession	Type	1) Homology arm sequence (LEFT HA, right HA; 5' → 3')<br>2) Sanger sequencing of mut allele after NHEJ (where applicable)	crRNA sequence (5' → 3')	Genotyping primers (5' → 3')	Genotyping PCR
ZDHHC12	Targeting construct: OM778729	KI/KI<br>KI/KI	1) GGGCGCTCCTCAGCCCTGGGGTCCTGGTGCGGACCGGGCACACCGTGCTGacctggggaatcacgc tggtgctcttcctgcacgataccggtgagccgga	CGGGCACACCGTGCTGACCT	Flanking (F):<br>Fwd: CTGTTGGCTTGAGGGTCCA<br>Rev: ACGTCCCCGCATGTTAGAAG (T2A-rev)<br><br>Spanning (S):<br>Fwd: CTGTTGGCTTGAGGGTCCA<br>Rev: CTCAAGTCGGTCCTTGGATC	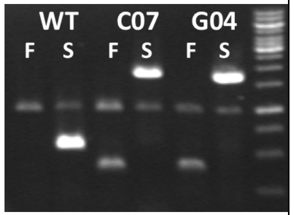
ZDHHC5	Targeting construct: OM778711<br><br>Mut allele (clone G06): OM778746	KI/mut	1) TGGTTTTCTTTTGTACTCCTCTCTGTTGCTTCCCTCCTCCCATTTTCTTGTCTGTTCTGCCGCTGTGT GGGCCTGGGCTATGCGGCAGGGCAGATTTCCCATCAGAGCTCCAACATGCCCGCAGAGTCTGG AAAGAGATTCAAACCCAGCaagtatgtcccggtctctgcagccgccatcttcctagtgggagctacgacactcttcttt gcctttacgtgagttttctcccagcaggggtgtttgggtgggtggatactccatgggaagtgaggagataacgctttctcttgag ttttg<br><br>2) mut allele sequence (clone G06)<br>TGTTCTGCCGCTGTGTGGGCCTGGGCTATGCGGCAGGGCAGATTTCCCATCAGAGCTCCAACAT GCCCGCAGAGTCTGGAAAGAGATTCAAACCCAGCAA[insertion:A]GTATGTCCCGGTCTCTGC AGCCGCCATCTTCCTAGTGGGAGCTACGACACTCTTCTTTGCCTTTACGTGAGTTTTCTCCCAGC AGGGGTGTTTGGGTGGG	GAGACCGGGACATACTTGCT	Flanking (F):<br>Fwd: ACCTGCCTCCATCCATGAG<br>Rev: ACGTCCCCGCATGTTAGAAG (T2A-rev)<br><br>Spanning (S):<br>Fwd: ACCTGCCTCCATCCATGAG<br>Rev: GTGACAGAGCAAGAGACGC	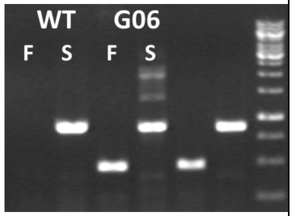

Table S02. Genes knocked out by out of frame/critical exon deletion or by truncation of functional domain.

**Gene**	**GenBank accession**	**Deletion**	**crRNA sequence (5' → 3')**	**Genotyping primers (5' → 3')**	**KO allele Sanger sequencing after NHEJ***	**Deleted sequence (5' → 3')**
ALDH3A2	KO allele, clone A06: OM778731<br><br>KO allele, clone B08: OM778732	Deletion of exon 2	Condition A<br>5' crRNA: GAATTAGCAAGCCCTTACCAGGG<br>3' crRNA: GGGCAGAGAGGACAATCTAAGGG<br><br>Condition B<br>5' crRNA: GTTGTCACTACAGGTGTACCTGG<br>3' crRNA: TATGAGAGTAATGCTAGCTTGGG	Flanking (F):<br>Fwd: AGTGTGTTCAGAGCAGACCTTTAT<br>Rev: AGCAGTAACCCATTCAGGAAGAT<br><br>Spanning (S):<br>Fwd: AGTGTGTTCAGAGCAGACCTTTAT<br>Rev: TGGGAAGAACAGGCTAGGTACT	Condition A, clone A06<br>TAAAGAAGGATTTACTAATGGTATTCGACTAGTGTTACTACTCCCTGAAATAATCTTTGATTTTTTTAAAAGAAATATATTAGAGAATTAGCAAGCCCTTagggcagaagtgggagaaaagcgatagaaagctggaggagtgggaaaggctgctggaacccattgtcagtgacctgaagatgaggacagctaaggagtta<br><br>Condition B, clone B08<br>AGAGAATTAGCAAGCCCTTACCAGGGGCGTGAAGGGTGCAAAAGGAGTCTGAATGGCAAACAGCTAGTCTGATAATGCCAGTTGTTGTCACTACAGGTGAcatcgctgcaggtctggtgccaccttatgtctatatacctttttagggaggcttattttctcatattaattggaaattaaggatagtggctaattaaata	Condition A, clone A06<br>accaggggcgtgaagggtgcaaaaggagtctgaatggcaaacagctagtctgataatgccagttgttgtcactacaggtgtacctggtgtgagtgttctgacattcagggccaagtgtatcatacttactctgcaagattaactgtgattctcttataacagagtgaattcaatgtgtacagtcaggaagtcattactgtccttggggaaattgattttatgcttgagaatcttcctgaatgggttactgctaaaccagttaagaagaacgtgctcaccatgctggatgaggcctatattcagccacagcctctgggagtggtgctgataatcggagcttggaattaccccttcgttctcaccattcagccactgataggagccatcgctgcaggtctggtgccaccttatgtctatatacctttttagggaggcttattttctcatattaattggaaattaaggatagtggctaattaaatacatttacttggtgatttgcctttgtttacaccaccagtgtactgagaattcatacataccatacatatttttaaatgggctgctgaggagatttgtgtggctatgttttgaagcacctggggaatgaacctgtgggagtgtaaagatgtgtgagtcagcaggcccaggagtttggagtcaggcatccatgttctaatatggtttcagttgctggctctggcttcggatgaaccatttgattctgtctgaccagtttcttcattcaaataaagactatgagagtaatgctagcttgggtatatagcaaatgccaggagattaatttctcactttgcaggatccagatagagagatgtatgttgaaatggctcacttgcccaataattccagggcagagaggacaatcta<br><br>Condition B, clone B08<br>acctggtgtgagtgttctgacattcagggccaagtgtatcatacttactctgcaagattaactgtgattctcttataacagagtgaattcaatgtgtacagtcaggaagtcattactgtccttggggaaattgattttatgcttgagaatcttcctgaatgggttactgctaaaccagttaagaagaacgtgctcaccatgctggatgaggcctatattcagccacagcctctgggagtggtgctgataatcggagcttggaattaccccttcgttctcaccattcagccactgataggagc

Gene	GenBank accession	Deletion	crRNA sequence (5’ → 3’)	Genotyping primers (5’ → 3’)	KO allele Sanger sequencing after NHEJ*	Deleted sequence (5’ → 3’)
CEPT1	KO allele, clone F10: OM778734<br><br>KO allele, clone B02: OM778733<br><br>KO allele, clone F04: OM778735	Out of frame deletion in exon 1	Condition A<br>5’ crRNA: TGAGTGGGCATCGATCAACAAGG<br>3’ crRNA: GTTCTTCTAGCCGCTTTAGTTGG<br><br>Condition B<br>5’ crRNA: TTCGGGCATATGAGTACTACAGG<br>3’ crRNA: TCGTTAGAAGAGTTCCCTCCTGG	Flanking (F):<br>Fwd: AGATGTAAATAGTGGCCAACAAGTG<br>Rev: ACAATGGTGGTGTTGGTAACTG<br><br>Spanning (S):<br>Fwd: AGATGTAAATAGTGGCCAACAAGTG<br>Rev: GCTCTGTAGCTGTAGGGCAG	Condition A, clones B08 and F10<br>TTTTTAGGTAAGCACCAGCCACAAAAACCTACAAAAGAAGGGAAATTACTGTCTTTAAATATTAAAAAAAAAACAAGATCCATGAGTGGGCATCGATCAA<br>aagcggctagaagaacacagatatcaaagtgctggacggtccctgcttgagcccttaatgcaagggtattgggaatggctcgttagaagagttccctcct<br><br>Condition B, clone B02<br>TTAAAAAAAAACAAGATCCATGAGTGGGCATCGATCAACAAGGAAAAGATGTGGAGATTCTCACCCGGAGTCCCCAGTGGGCTTCGGGCATATGAGTACTcctggattgccccaaatctcatcaccatcattggactgtcaataaacatctgtacaactattttattagtcttctactgccctacagctacagagcaggt<br><br>Condition B, clone F04<br>ATATTAAAAAAAACAAGATCCATGAGTGGGCATCGATCAACAAGGAAAAGATGTGGAGATTCTCACCCGGAGTCCCCAGTGGGCTTCGGGCATATGAGTAggattgccccaaatctcatcaccatcattggactgtcaataaacatctgtacaactattttattagtcttctactgccctacagctacagagcaggtaag	Condition A, clones B08 and F10<br>caaggaaaagatgtggagattctcacccggagtccccagtgggcttcgggcatatgagtactacaggatgtgtattaaataaattgtttcagttaccaacaccaccattgtcaagacaccaacta<br><br>Condition B, clone B02<br>acaggatgtgtattaaataaattgtttcagttaccaacaccaccattgtcaagacaccaactaaagcggctagaagaacacagatatcaaagtgctggacggtccctgcttgagcccttaatgcaagggtattgggaatggctcgttagaagagttccct<br><br>Condition B, clone F04<br>ctacaggatgtgtattaaataaattgtttcagttaccaacaccaccattgtcaagacaccaactaaagcggctagaagaacacagatatcaaagtgctggacggtccctgcttgagcccttaatgcaagggtattgggaatggctcgttagaagagttccctcct
CERS2	KO allele, clone A10: OM778736<br><br>KO allele, clone B11: OM778737	Deletion of exon 2	Condition A<br>5’ crRNA: TGTGTTCACTAGTGGCCAGGCGG<br>3’ crRNA: TGCTGACTGAGGTTGTTACTGGG<br><br>Condition B<br>5’ crRNA: ACTAGTGGCCAGGCGGGCGGCGG<br>3’ crRNA: GGGATGCTGCTGAGTTCCGAAGG	Flanking (F):<br>Fwd: TGCCAGTTGCAGTTTCTTGG<br>Rev: CGTACCTGGGGAAGGGATATG<br><br>Spanning (S):<br>Fwd: TGCCAGTTGCAGTTTCTTGG<br>Rev: ACTTCTTGAGGAGACTGGGC	Condition A, clone A10:<br>CCCTCCCCTGAAGGCACCATTGTCTTCCTGAAGGCTCAGCACCCCCAGGCCCTTTCCATCAATGGTTATTTGTTCTCACCCTCTACCCGCCGCCCGCCTGactgggagaggctggggatgctgctgagttccgaaggtaaacataacaatgggcttcttcactgtgcccaggtggaagtagagcttttgtcccggcagag<br><br>Condition B, Clone B11:<br>GTGCACCCCCTCCCCTGAAGGCACCATTGTCTTCCTGAAGGCTCAGCACCCCCAGGCCCTTTCCATCAATGGTTATTTGTTCTCACCCTCTACCCGCCGCgaaggtaaacataacaatgggcttcttcactgtgcccaggtggaagtagagcttttgtcccggcagagcgggctctctggccgccaggtagagcgttggt	Condition A, clone A10:<br>gccactagtgaacacacaggcagctttgtcagctgtgggcagccctggaggctagagaaaagggggttgccaggaagctggggaaactgaggccagggtaaagttgccttagcttgcagccctttcccttcccagcaagctagataccttactcatatcccttccccaggtacgtggctacaccactggctgccctcttgaacataaaggagaaaactcggctgcgggcacctcccaacgccaccttggaacatttctacctgaccagtggcaagcagcccaagcaggtatgagccgcatgctgctctggctctgggaatcactgagtttggtggtgggggacagggtttgaatgttagctctggcaggtgaagagatggggaagcagtggtgctgactgaggttgtt<br><br>Condition B, Clone B11<br>ccgcctggccactagtgaacacacaggcagctttgtcagctgtgggcagccctggaggctagagaaaagggggttgccaggaagctggggaaactgaggccagggtaaagttgccttagcttgcagccctttcccttcccagcaagctagataccttactcatatcccttccccaggtacgtggctacaccactggctgccctcttgaacataaaggagaaaactcggctgcgggcacctcccaacgccaccttggaacatttctacctgaccagtggcaagcagcccaagcaggtatgagccgcatgctgctctggctctgggaatcactgagtttggtggtgggggacagggtttgaatgttagctctggcaggtgaagagatggggaagcagtggtgctgactgaggttgttactgggagaggctggggatgctgctgagttcc

Gene	GenBank accession	Deletion	crRNA sequence (5' → 3')	Genotyping primers (5' → 3')	KO allele Sanger sequencing after NHEJ*	Deleted sequence (5' → 3')
FADS3	KO allele, clone C02: OM778738	Deletion of exons 4 and 5 (truncation of FA desaturase)	Condition A<br>5' crRNA: TGAAATGGAGCATTAGCACGGGG<br>3' crRNA: TGGGGCGGGGCACGTCTGTCAGG<br><br>Condition B<br>5' crRNA: GAAATGGAGCATTAGCACGGGGG<br>3' crRNA: TGACAGACGTGCCCCGCCCCAGG	Flanking (F):<br>Fwd: AGTGAACAGTCTGAAGGCCC<br>Rev: TTTAGCTGCCCCATCACGAA<br><br>Spanning (S):<br>Fwd: AGTGAACAGTCTGAAGGCCC<br>Rev: CCAAAGACTCTAGGGCTCCG	Condition B, clone C02:<br>GCCCTGCAGCTTCATGACACTTACGAGCCCCTCCACC<br>TCCCTGGGACTCAGTTCTCATCTGTAAAAAGAGGACA<br>CTGGCCCACAAGGGTCTTGAAATGGAgccccagccagcc<br>ccgtgaccttgcccgggagaaggaggtggcctggagagctgctgtct<br>ccagccgccgcctgtctccacagtatggcaagaagaaacg	Condition B, clone C02:<br>gcattagcacgggggtaccctgcaagctgaaaggattcactggggccccagg<br>ccctggcgggctccgtccttcccaacagcttctgaccctgcctctccccagg<br>ctcagtcctggtgtctgcagcatgacctgggccatgcctccatcttcaagaagt<br>cctggtggaaccacgtggcccagaagttcgtgatggggcagctaaaggtgag<br>ggtggggtgggtggtcagccaggtgctgggtggcgctgggtctgcccaagtgt<br>gtgggcacagtcgggggcacagcctgccctgagagccccctcctcctccaca<br>gggcttctccgcccactggtggaacttccgccacttccagcaccacgccaagc<br>ccaacatcttccacaaagacccagacgtgacggtggcgcccgtcttcctcctg<br>ggggagtcatccgtcgaggtgggtggggagggacgtggacaacctctggctg<br>ggcctgcagctgagggggagctaatgcactgggtccccactctgcccctgacc<br>tagcccctgatctggcctccactctggctgggccaagctctgcccccgtgtcttt<br>ccttcccacctcccaacctgctggggacgaccagcccgcttgctagaatctag<br>agttgcctttgacccttg
GNPAT	KO allele, clone A10: OM778739	Deletion of exon 4	Condition A<br>5' crRNA: GATGGCCTGAGGAGTATATTTGG<br>3' crRNA: GGCATATTCTAGCAGTACTGAGG<br><br>Condition B<br>5' crRNA: GAGCAAATCGTTTATGTGTATGG<br>3' crRNA: AAGTTATTCAGTGCTAGGACTGG	Flanking (F):<br>Fwd: CTGCTGCCTAGTCATCGAAGT<br>Rev: TTATTGCCTAGAGAGGTAGCCAT<br><br>Spanning (S):<br>Fwd: ACTGACTGTTTTCGAGATTGCTG<br>Rev: TTATTGCCTAGAGAGGTAGCCAT	Condition A, clone A10<br>AGATTGCTGACTTATACCCTGAATCTGTCAGAGAATG<br>TTACAGTGTTTGGGACCAAGGGTGGTGGTGAGCCAT<br>TTCTTAAGTGATGATCACAGATTACCTggtatttgaagatg<br>atgatgaaaatagcctctcctcctttcttccagtcctagcactgaataa<br>cttgagttatgtcctttgtcctgtgctttactgtgcc	Condition A, clone A10<br>ggcctctgttacatttagatttcaaaaaaacagataacacatggataccagtg<br>aatcttgagattgatggcctgaggagtatatttggaaactcaaattatccctcc<br>cagccacatgacacccattcaccctggcagtgagtccagcacatagagaaat<br>accattcttctcaccttgtcctatgaatttgtagattttttttaatctctccaagag<br>aagctttgttttctcttactacaaccaaccaattcaatacagtggttaacagttg<br>gctttttaagtcagactgacttgggttccattcacagctctgccatttattggat<br>gcatgaccattttacaaacaagaaagctaaggcttagaccttgttaattatgtg<br>gtattaaacaaagcacgtaagataactgtatataaaatgatgctaatatagtg<br>cccaactaatatatgttagcgatcatcactgttattattctgatccataaaaatt<br>tacatttttaatattcttgcattatgtgaaattgtttaactagaaacagtactttt<br>accttatcagagcaaatcgtttatgtgtatggcaaaaaggcagaaaaagagg<br>atgggtattccctctaaatatagacttattcttccgttttctgatttgagatgtga<br>tttgataacatgacaactgaaattattcaaaatgtaacatttactttcaagcta<br>caaagagccatccaggagcatcctgttgttctgctgcctagtcatcgaagttac<br>attgacttcctcatgttgtcttttcttctatacaattatgatttgcctgtgccagtt<br>atagcagcaggaatgggtatgtattgtttttctgtttttttaactgtaaaaatta<br>aaaagttcactggcatattctagcagtactga

* The sequence includes 100 bp upstream (upper case) and 100 bp downstream (lower case) of the NHEJ site.

Gel originals

Original, unprocessed versions of the gels presented in **Table S01**.

ACOT7, SAMD8 and KMT2C

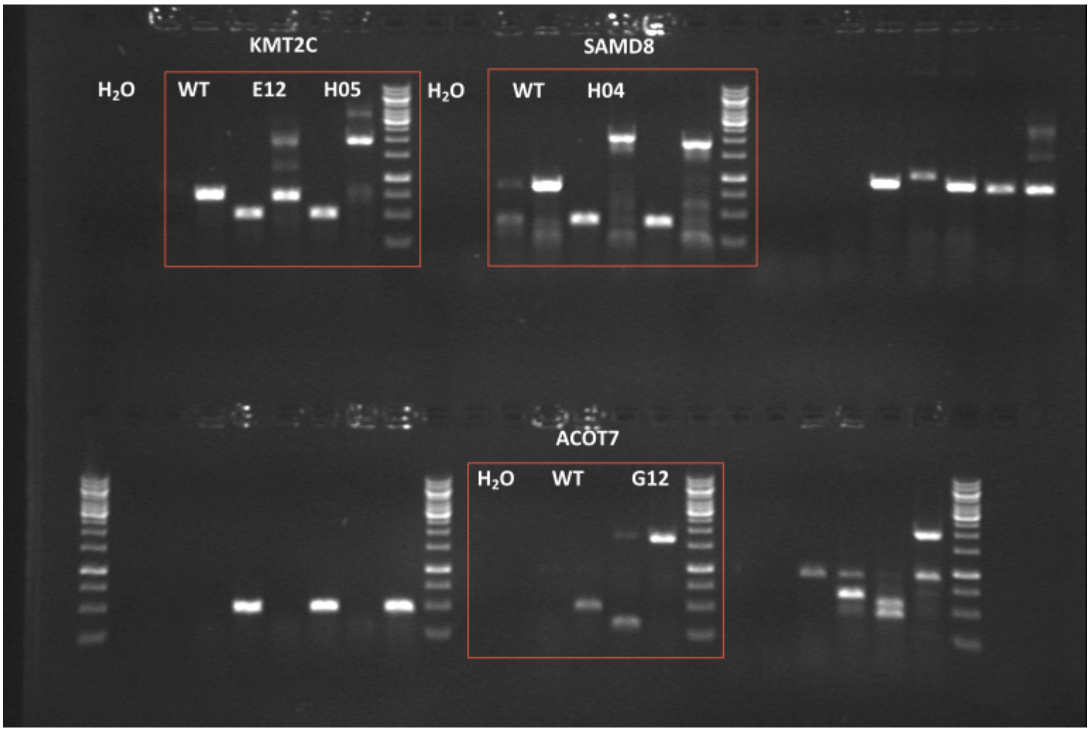

ACSL3, ZDHHC5, and THUMPD3

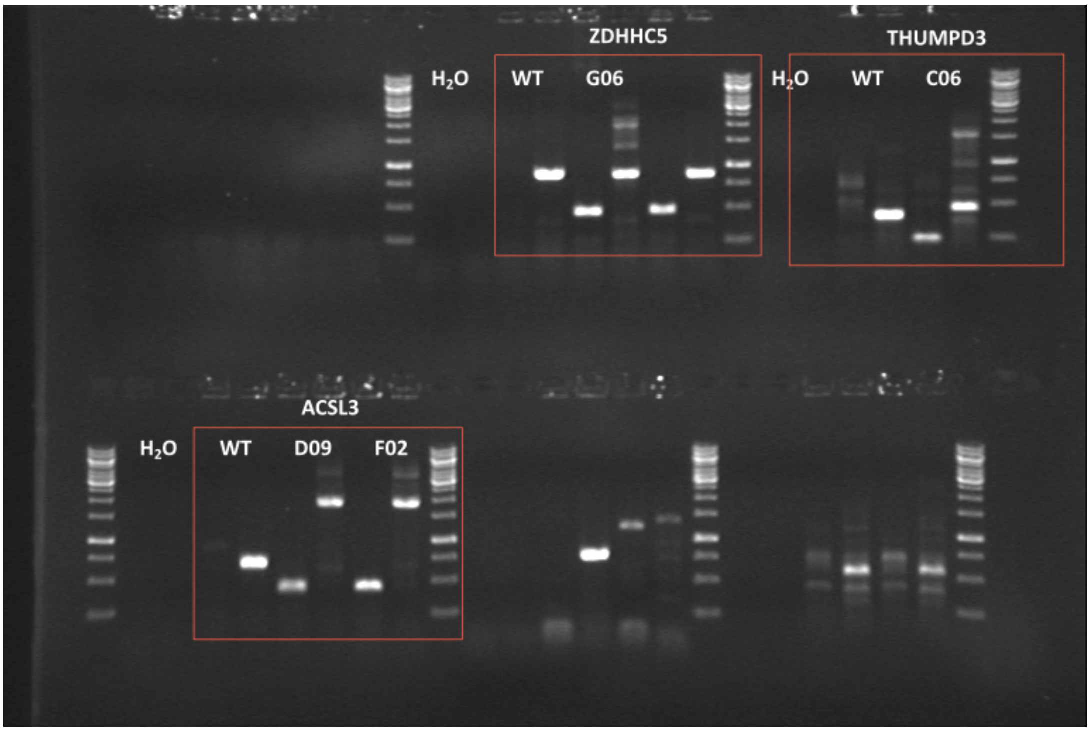

CEPT1, ELOVL5, GNPAT, and SGMS1

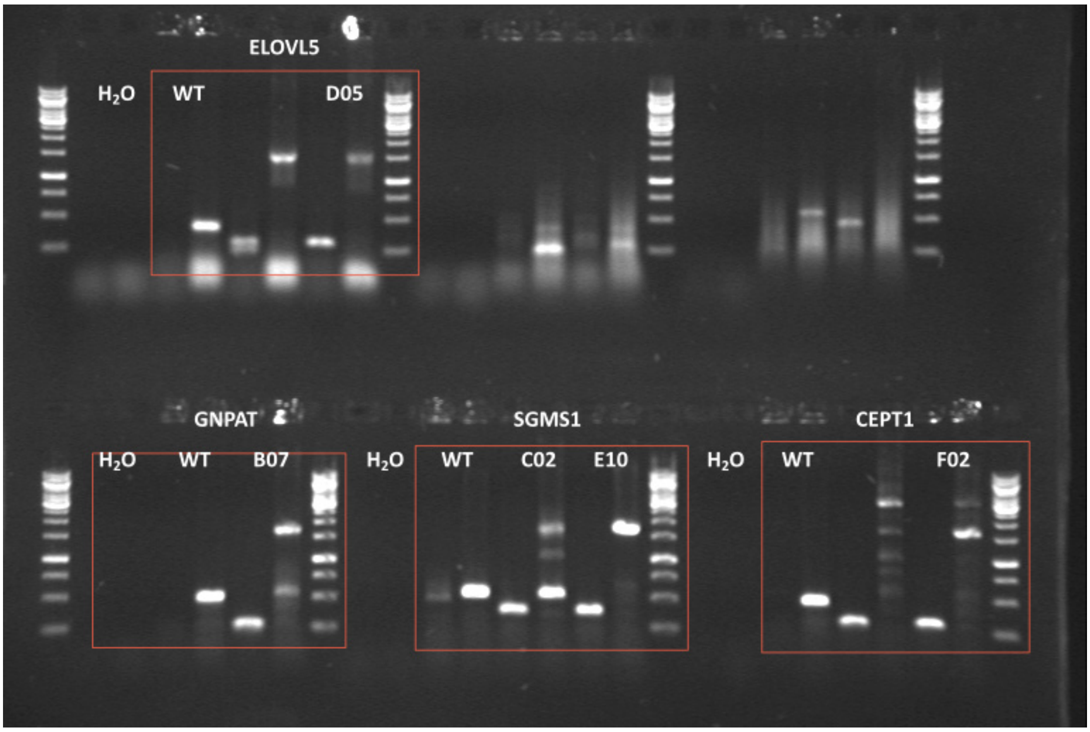

DECR2

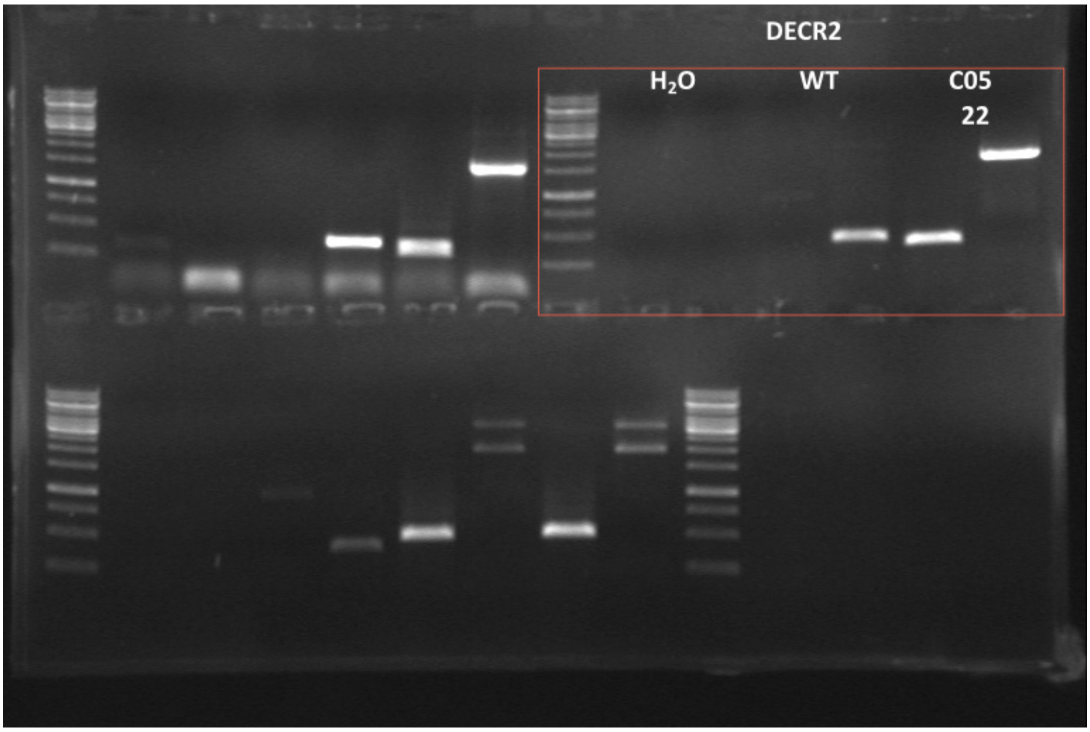

DLAT, NSUN3, HSD17B12, and PLD3

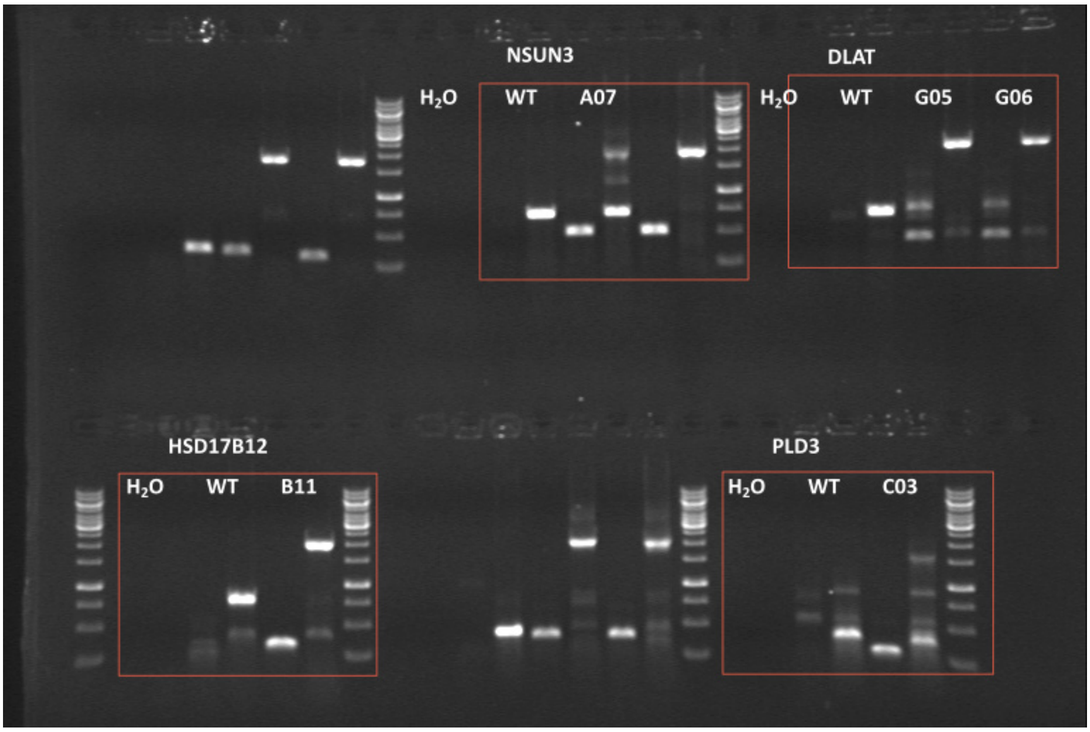

LPIN1

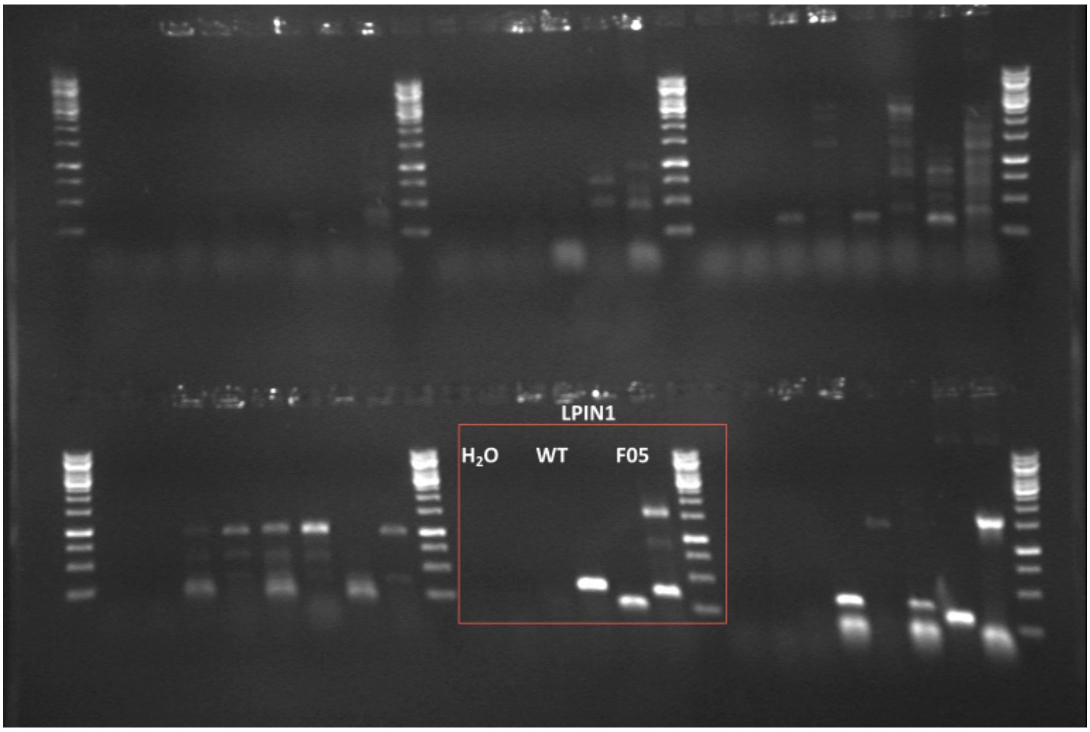

MPDU1 and NSUN2

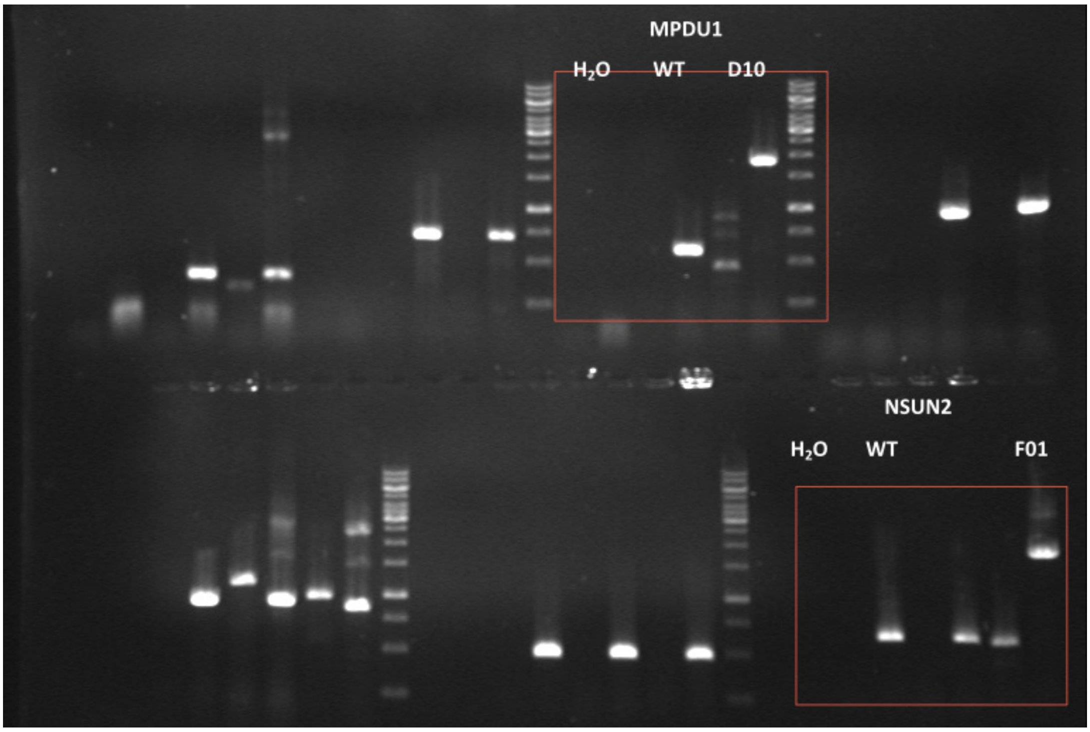

ORMDL2

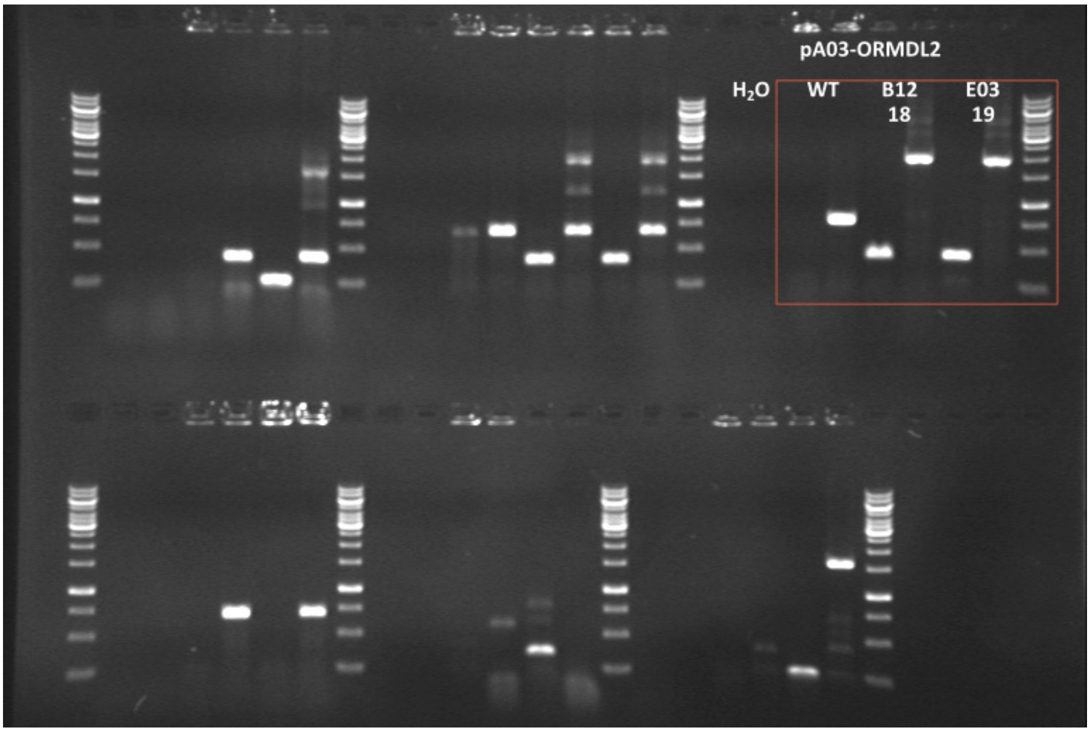

ZDHHC12

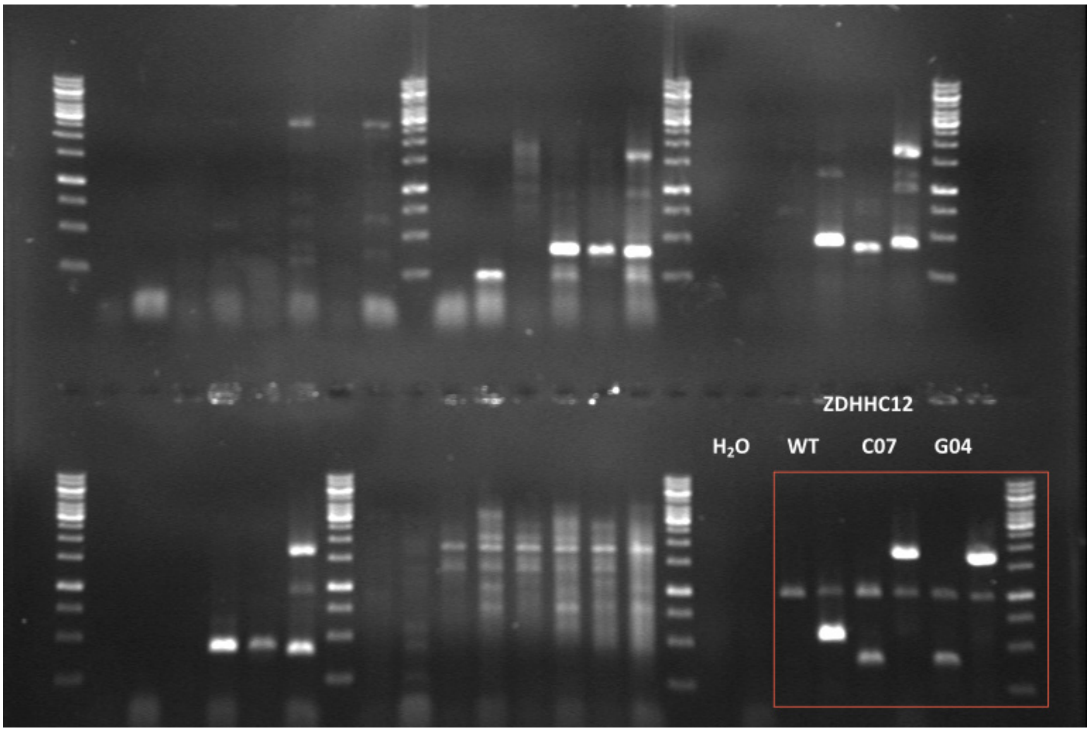

References

Zhu, Xiphias Ge, Shirony Nicholson Puthenveedu, Yihui Shen, Konnor La, Can Ozlu, Tim Wang, Diana Klompstra, et al. 2019. "Chp1 Regulates Compartmentalized Glycerolipid Synthesis by Activating Gpat4." *Molecular Cell* 74 (1): 45–58.e7. https://doi.org/10.1016/j.molcel.2019.01.037.
